# Evaluating the electronic structure of formal Ln^II^ ions in Ln^II^(C_5_H_4_SiMe_3_)_3_^1–^ using XANES spectroscopy and DFT calculations†
†Electronic supplementary information (ESI) available: The results from quantum chemical *ab initio* FEFF9.6 code calculations for Ln^II^ L_3_-edges from Ln(C_5_H_4_SiMe_3_)_3_^*x*–^ (Ln = Sm, Tm, Y; *x* = 0, 1) and second derivative analysis of the lanthanides are in the ESI.† Our branching ratio analysis, the PBE//TZP optimized ground-state geometrical *xyz* coordinates and XC//TZ2P (XC = PBE, BLYP, B3LYP, BHandHLYP), single-point calculated total bonding energies of Ln(C_5_H_4_SiMe_3_)_3_^1–/0^ (Ln = Sm, Ho), and relative single-point energy difference in kcal mol^–1^ between 4f^10^ 5d^1^ and 4f^11^ 5d^0^ in Ho^II^(C_5_H_4_SiMe_3_)_3_^1–^ from different functional results at the PBE//TZP optimized ground-state geometries are also included. See DOI: 10.1039/c7sc00825b



**DOI:** 10.1039/c7sc00825b

**Published:** 2017-06-30

**Authors:** Megan E. Fieser, Maryline G. Ferrier, Jing Su, Enrique Batista, Samantha K. Cary, Jonathan W. Engle, William J. Evans, Juan S. Lezama Pacheco, Stosh A. Kozimor, Angela C. Olson, Austin J. Ryan, Benjamin W. Stein, Gregory L. Wagner, David H. Woen, Tonya Vitova, Ping Yang

**Affiliations:** a University of California , Irvine , CA 92697 , USA . Email: wevans@uci.edu; b Los Alamos National Laboratory , Los Alamos , NM 87545 , USA . Email: stosh@lanl.gov ; Email: pyang@lanl.gov ; Email: erb@lanl.gov; c University of Wisconsin , Madison , Wisconsin 53711 , USA; d Stanford University , Palo Alto , CA 94305 , USA; e Karlsruhe Institute of Technology , Institute for Nuclear Waste Disposal , P.O. Box 3640 , 76021 Karlsruhe , Germany

## Abstract

The isolation of [K(2.2.2-cryptand)][Ln(C_5_H_4_SiMe_3_)_3_], formally containing Ln^II^, for all lanthanides (excluding *Pm*) was surprising given that +2 oxidation states are typically regarded as inaccessible for most 4f-elements. Herein, X-ray absorption near-edge spectroscopy (XANES), ground-state density functional theory (DFT), and transition dipole moment calculations are used to investigate the possibility that Ln(C_5_H_4_SiMe_3_)_3_^1–^ (Ln = Pr, Nd, Sm, Gd, Tb, Dy, Y, Ho, Er, Tm, Yb and Lu) compounds represented molecular Ln^II^ complexes. Results from the ground-state DFT calculations were supported by additional calculations that utilized complete-active-space multi-configuration approach with second-order perturbation theoretical correction (CASPT2). Through comparisons with standards, Ln(C_5_H_4_SiMe_3_)_3_^1–^ (Ln = Sm, Tm, Yb, Lu, Y) are determined to contain 4f^6^ 5d^0^ (Sm^II^), 4f^13^ 5d^0^ (Tm^II^), 4f^14^ 5d^0^ (Yb^II^), 4f^14^ 5d^1^ (Lu^II^), and 4d^1^ (Y^II^) electronic configurations. Additionally, our results suggest that Ln(C_5_H_4_SiMe_3_)_3_^1–^ (Ln = Pr, Nd, Gd, Tb, Dy, Ho, and Er) also contain Ln^II^ ions, but with 4f^*n*^ 5d^1^ configurations (not 4f^*n*+1^ 5d^0^). In these 4f^*n*^ 5d^1^ complexes, the *C*_3h_-symmetric ligand environment provides a highly shielded 5d-orbital of *a*′ symmetry that made the 4f^*n*^ 5d^1^ electronic configurations lower in energy than the more typical 4f^*n*+1^ 5d^0^ configuration.

## Abstract 2

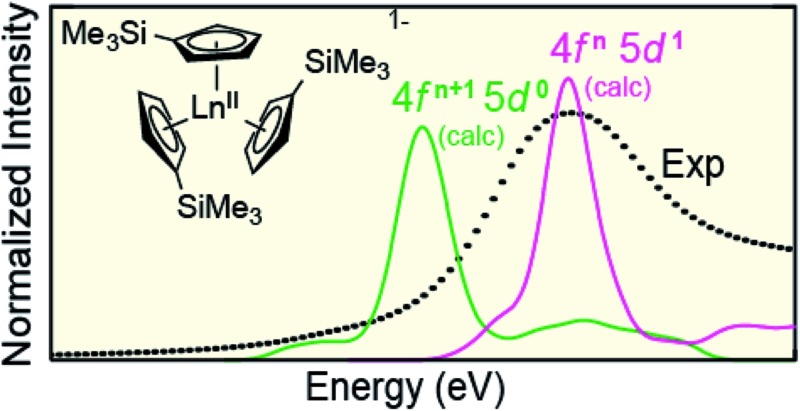
Ln^II^(C_5_H_4_SiMe_3_)^1–^ have been characterized by XANES and DFT.

## Introduction

**Scheme 1:**
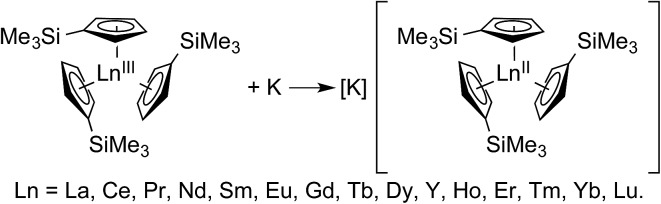
A general reaction scheme for generating Ln^II^(C_5_H_4_SiMe_3_)_3_^1–^ containing salts. Accessing these compounds in crystalline form requires complexation of the potassium cation by 18-crown-6 or 2.2.2-cryptand.^1^

Recent advances in rare-earth metal reduction chemistry have revealed a surprisingly new series of molecular complexes that contained all the rare earth metals in the formal oxidation state of +2,^1^ as defined by Parkin and Karen, (Scheme 1).^2,3^ These results were unexpected given that the +2 oxidation state had only been observed with six rare earth metals in molecules (Eu, Yb, Sm, Tm, Dy, and Nd). Observing this +2 oxidation state for the other lanthanides was unexpected because the –2.7 to –3.9 V *versus* standard hydrogen electrode (SHE) Ln^III^/Ln^II^ reduction potentials seemed too negative to allow Ln^II^ ions to exist in solution.^4^ In the solid state, only the six lanthanides listed above were known to form +2 salts. For the other metals, compounds like LnX_2_ (Ln = La, Ce, Pr, Gd, and Y; X = halide) with formal +2 oxidation states had been observed, but subsequent analyses revealed that they contain +3 ions and a delocalized electron in a conduction band, *i.e.* Ln^III^(X^1–^)_2_(e^1–^).^5^

The new Ln(C_5_H_4_SiMe_3_)_3_^1–^ compounds, containing the putative +2 ions, were synthesized by potassium reduction of trimethylsilylcylopentadienyl lanthanide(iii) complexes, Ln(C_5_H_4_SiMe_3_)_3_ (Scheme 1). More detailed synthetic descriptions for these Ln(C_5_H_4_SiMe_3_)_3_^1–^ anions, as well as related Ln[C_5_H_3_(SiMe_3_)_2_]_3_^1–^ complexes prepared by Lappert and coworkers, have been previously discussed.^6^ The new Ln(C_5_H_4_SiMe)_3_^1–^ complexes were unusual in that their Ln–C_centroid_ distances were only 1% (0.020–0.032 Å) longer than their Ln^III^ precursors, Ln(C_5_H_4_SiMe_3_)_3_. Larger variations, by an order of magnitude (0.1 to 0.2 Å), were expected based on previous comparisons between conventional Ln^II^ *versus* Ln^III^ structures, which historically provided a diagnostic for the +2 oxidation state. Consistent with this traditional expectation, Ln(C_5_H_4_SiMe_3_)_3_^1–^ bond lengths for Ln = Eu, Yb, Sm, and Tm were 0.10–0.20 Å (∼6%) longer than their +3 analogs.^7^ The unusually short bond lengths in the La, Ce, Pr, Nd, Gd, Tb, Dy, Y, Ho, Er, and Lu complexes led to skepticism about the presence of the +2 oxidation state across the Ln(C_5_H_4_SiMe_3_)_3_^1–^ series, suggesting that the salts might contain +3 metals with an electron delocalized into ligand-based orbitals. This scenario was – in a sense – reminiscent of the LnX_2_ compounds (discussed above).^5^ An alternative description, based on subsequent theoretical analyses, proposed that the small differences in bond distances for La, Ce, Pr, Nd, Gd, Tb, Dy, Y, Ho, Er, and Lu complexes were a direct result of the metal ions having an unusual 4f^*n*^ 5d^1^ electronic configuration, rather than the traditionally expected 4f^*n*+1^ 5d^0^ configuration known for Eu^II^, Yb^II^, Sm^II^, and Tm^II^.

Attempts have been made to validate the theoretical conclusions using electronic absorption spectroscopy and magnetic susceptibility.^8^ Although the UV-vis analyses showed intense bands that were consistent with the 4f^*n*^ 5d^1^ configurations, forbidden 4f → 4f transitions typically used as diagnostics for lanthanide oxidations states were not experimentally resolved.^1,5,9^ Similarly, the magnetic studies showed complicated magnetic behavior that could not be ubiquitously rationalized for all the lanthanides using simple models.^8*d*^ For these reasons, it was of great interest to evaluate the electronic structure of the Ln(C_5_H_4_SiMe_3_)_3_^1–^ complexes using a combination of X-ray absorption near-edge spectroscopy (XANES) and transition dipole moment density functional theory (DFT). There is an emerging body of literature demonstrating the power of cooperative XANES and DFT analyses in evaluating bonding and electronic structure in inorganic compounds.^10^ As such, we have recently used this approach to uniquely characterize the electronic structures of a wide variety of f-element species.^11^

**Scheme 2:**
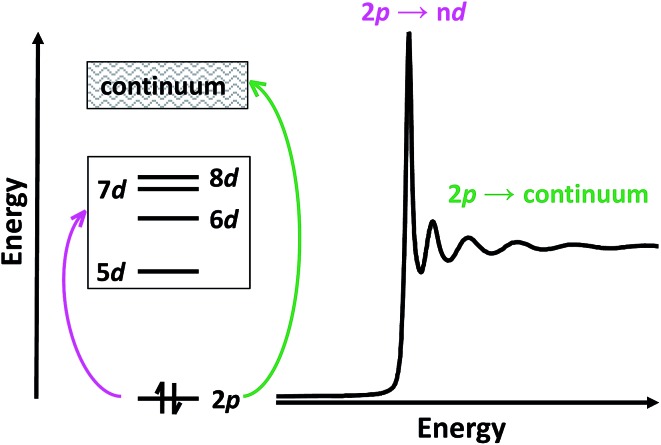
Cartoon depicting the origin of L_3_-edge XANES transitions.

Herein, we describe the use of a combination of XANES and transition dipole moment DFT calculations to evaluate the possibility that the Ln^II^(C_5_H_4_SiMe_3_)_3_^1–^ (Ln = Pr, Nd, Sm, Gd, Tb, Dy, Y, Ho, Er, Tm, Yb and Lu) compounds represent molecular Ln^II^ complexes. In the XANES experiment, an analyte is exposed to high-energy X-rays that excite core electrons to higher, unoccupied states. At the Ln L_3,2_-edges, there is an edge-jump consisting of electric-dipole allowed transitions from Ln 2p-orbitals to unoccupied states that contain metal d-character. Moving to higher energies, core electrons are excited into the continuum (Scheme 2). Given that Ln L_3,2_-edge XANES probes transitions to Ln 5d-orbitals, this spectroscopic approach provides a particularly sensitive and accurate method for directly characterizing 5d-orbital occupancies for the alleged 4f^*n*^ 5d^1^ ions in Ln(C_5_H_4_SiMe_3_)_3_^1–^ (Ln = La, Ce, Pr, Nd, Gd, Tb, Dy, Ho, Er, and Lu) anions. To guide interpretations of these XANES spectra, appropriate ground-state DFT models were developed that formed a basis for extracting probability amplitudes from the transition dipole moments between the calculated excited-states and the ground-state. Combined, these computational and experimental efforts allow the influence of 4f^*n*+1^ 5d^0^ *versus* 4f^*n*^ 5d^1^ electronic configurations on the lanthanide L_3_-edge XANES spectra to be determined for the first time.

To best characterize the electronic structure of the [K(2.2.2-cryptand)][Ln(C_5_H_4_SiMe_3_)_3_] salts containing new Ln^II^ ions, XANES and DFT studies are also reported with the compounds containing traditional +2 ions (*i.e.* Sm^II^, Tm^II^, and Yb^II^) whose electronic configurations were well defined as 4f^6^, 4f^13^, and 4f^14^, respectively. These results provide a foundation for analyses of the other Ln(C_5_H_4_SiMe_3_)_3_^1–^ anions. For comparison, studies of the neutral 4f^n^ 5d^0^ Ln^III^ complexes, Ln(C_5_H_4_SiMe_3_)_3_, are also reported because the metal oxidation state in these compounds is unambiguously +3. These combined efforts lead to a definitive description of the electronic structure and bonding in the Ln(C_5_H_4_SiMe_3_)_3_^1–^ complexes. For the convenience of the reader in the rest of the paper, we refer to compounds with formal +3 oxidation states as Ln^III^(C_5_H_4_SiMe_3_)_3_ and formal +2 oxidation states as Ln^II^(C_5_H_4_SiMe_3_)^1–^. When discussing both, the Roman numerals are omitted and Ln(C_5_H_4_SiMe_3_)_3_^*x*–^ (*x* = 0, 1) is used.

## Results

### Sm L_3,2_-edge XANES

**Fig. 1:**
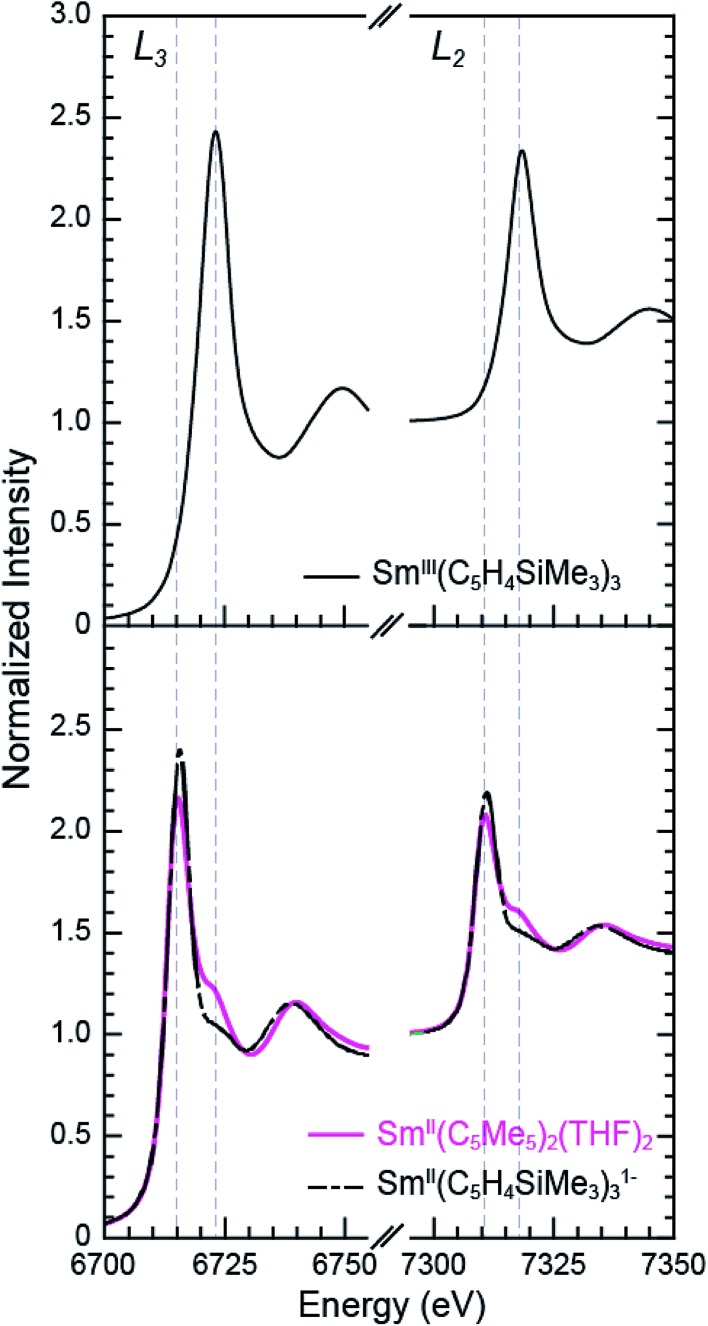
The background-subtracted and normalized Sm L_3,2_-edge XANES spectra obtained from Sm^III^(C_5_H_4_SiMe_3_)_3_ (top, black trace), Sm^II^(C_5_Me_5_)_2_(THF)_2_ (bottom, pink trace), and [K(2.2.2-cryptand)][Sm^II^(C_5_H_4_SiMe_3_)_3_] (bottom, black dashed trace).

The background-subtracted and normalized Sm L_3,2_-edge XANES spectra from [K(2.2.2-cryptand)][Sm^II^(C_5_H_4_SiMe_3_)_3_] and Sm^II^(C_5_Me_5_)_2_(THF)_2_ are shown in Fig. 1. Each spectrum contains large edge features near 6715 eV (L_3_) and 7310 eV (L_2_) and small post-edge shoulders near 6725 and 7320 eV that are superimposed on step-like absorption thresholds. The L_3,2_-edge positions were characterized by their peak maxima, where the first derivatives of the data equaled zero (Table 1). Given the sharp characteristics of these peaks, we find that the peak maximum provides a more useful metric than the inflection point, which is commonly used to evaluate actinide absorption edges. The L_3,2_-edge peak maxima for Sm^II^(C_5_H_4_SiMe_3_)_3_^1–^ at 6715.6 and 7311.1 eV are nearly identical to the 6715.2 and 7310.7 eV values determined for Sm^II^(C_5_Me_5_)_2_(THF)_2_ and similar to the other Sm^II^ L_3,2_-edge XANES spectra reported previously (Table 1).^12^

**Table 1:** Comparison of the peak maxima for Ln^III^(C_5_H_4_SiMe_3_)_3_, [K(2.2.2-cryptand)][Ln^II^(C_5_H_4_SiMe_3_)_3_] (Ln = Pr, Nd, Sm, Gd, Tb, Dy, Y, Ho, Er, Tm, Yb and Lu), Sm^II^(C_5_Me_5_)_2_(THF)_2_, Sm^II^[N(SiMe_3_)_2_](THF)_2_, Sm^III^[N(SiMe_3_)_2_]_3_, TmI_2_(THF)_3_, and TmI_3_(THF)_3.5_. When possible, spectral differences between analogous Ln^II^ and Ln^III^ compounds have been included

Compound	Edge	Peak position (eV)^*a*^	Δ(Ln^III^–Ln^II^) peak position (eV)
Pr^II^(C_5_H_4_SiMe_3_)_3_^1–^	L_2_	6444.5	0.2
Pr^III^(C_5_H_4_SiMe_3_)_3_	L_2_	6444.7	
Nd^II^(C_5_H_4_SiMe_3_)_3_^1–^	L_2_	6728.5	0.3
Nd^III^(C_5_H_4_SiMe_3_)_3_	L_2_	6728.8	
Sm^II^(C_5_H_4_SiMe_3_)_3_^1–^	L_3_	6715.6	7.6
	L_2_	7311.1	7.3
Sm^III^(C_5_H_4_SiMe_3_)_3_	L_3_	6723.2	
	L_2_	7318.4	
Sm^II^[N(SiMe_3_)_2_](THF)_2_	L_3_	6715.0	7.8
Sm^III^[N(SiMe_3_)_2_]_3_	L_3_	6722.8	
Sm^II^(C_5_Me_5_)_2_(THF)_2_	L_3_	6715.2	—
	L_2_	7310.7	
Gd^II^(C_5_H_4_SiMe_3_)_3_^1–^	L_3_	7248.6	0.3
Gd^III^(C_5_H_4_SiMe_3_)_3_	L_3_	7248.9	
Tb^II^(C_5_H_4_SiMe_3_)_3_^1–^	L_3_	7520.3	0.9
	L_2_	8258.1	1.0
Tb^III^(C_5_H_4_SiMe_3_)_3_	L_3_	7521.2	
	L_2_	8259.1	
Dy^II^(C_5_H_4_SiMe_3_)_3_^1–^	L_3_	7798.1	0.4
Dy^III^(C_5_H_4_SiMe_3_)_3_	L_3_	7798.5	
Ho^II^(C_5_H_4_SiMe_3_)_3_^1–^	L_3_	8075.6	0.5
	L_2_	8922.3	0.3
Ho^III^(C_5_H_4_SiMe_3_)_3_	L_3_	8076.1	
	L_2_	8922.6	
Er^II^(C_5_H_4_SiMe_3_)_3_^1–^	L_3_	8364.0	0.5
Er^III^(C_5_H_4_SiMe_3_)_3_	L_3_	8364.5	
Tm^II^(C_5_H_4_SiMe_3_)_3_^1–^	L_3_	8647.5	7.0
	L_2_	9617.1	6.6
Tm^III^(C_5_H_4_SiMe_3_)_3_	L_3_	8654.5	
	L_2_	9623.7	
Tm^II^I_2_(THF)_3_	L_3_	8646.3	7.7
	L_2_	9616.0	7.0
Tm^III^I_3_(THF)_3.5_	L_3_	8653.8	
	L_2_	9623.0	
Yb^II^(C_5_H_4_SiMe_3_)_3_^1–^	L_3_	8942.7	7.3
Yb^III^(C_5_H_4_SiMe_3_)_3_	L_3_	8950.0	
Lu^II^(C_5_H_4_SiMe_3_)_3_^1–^	L_3_	9244.4	1.9
Lu^III^(C_5_H_4_SiMe_3_)_3_	L_3_	9246.3	
Y^II^(C_5_H_4_SiMe_3_)_3_^1–^	K	17 052.6,^*a*^ 17 047.3^*b*^	1.0
Y^III^(C_5_H_4_SiMe_3_)_3_	K	17 053.6,^*a*^ 17 048.7^*b*^	

^*a*^The peak position points were defined as the first point at which the first derivative of the data equaled zero.

^*b*^Because the yttrium measurements were made at the Y K-edge, inflection points for Y^III^(C_5_H_4_SiMe_3_) and [K(2.2.2-cryptand)][Y^II^(C_5_H_4_SiMe_3_)] are reported.

**Fig. 2:**
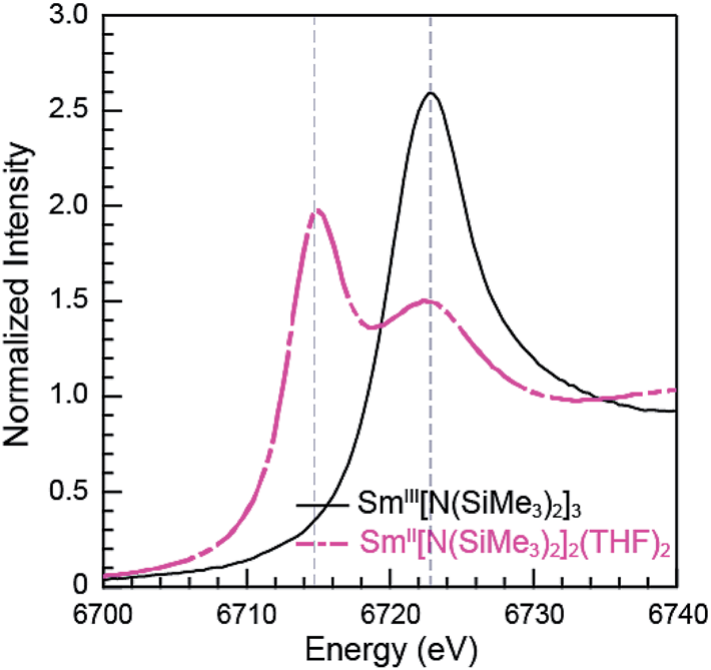
The background-subtracted and normalized Sm L_3_-edge XANES measurements obtained from the Sm^III^[N(SiMe_3_)_2_]_3_ (black trace) and Sm^II^[N(SiMe_3_)_2_]_2_(THF)_2_ (pink dashed trace).

The Sm L_3,2_-edge XANES spectra obtained from Sm^II^(C_5_H_4_SiMe_3_)_3_^1–^ and Sm^II^(C_5_Me_5_)_2_(THF)_2_ are also compared with Sm^III^(C_5_H_4_SiMe_3_)_3_ in Fig. 1. The Sm^III^(C_5_H_4_SiMe_3_)_3_ L_3,2_-edge spectra differ from the Sm^II^ spectra in that the edge features are shifted by approximately 7–8 eV to higher energies at 6723.2 and 7318.4 eV, Table 1. The differences in edge-positions for 4f^6^ 5d^0^ (+2) and 4f^5^ 5d^0^ (+3) samarium species are not unique to this suite of samarium cyclopentadienyl compounds.^10,13^ For instance, the Sm L_3_-edge XANES spectra obtained from Sm^II^[N(SiMe_3_)_2_]_2_(THF)_2_ and Sm^III^[N(SiMe_3_)_2_]_3_, also exhibit a Sm L_3_-edge energy difference of 7–8 eV (Fig. 2 and Table 1). These results demonstrate that samarium 4f-orbital occupancy (4f^6^ 5d^0^ *versus* 4f^5^ 5d^0^) influences the peak position more substantially than the ligand identity, as changing cyclopentadienide in Sm^III^(C_5_H_4_SiMe_3_)_3_ to amido ligands in Sm^III^[N(SiMe_3_)_2_]_3_ only shifts the L_3_-edge peak maximum to lower energy by 0.4 eV.

Comparisons between the Sm^II^ and Sm^III^ spectra provide insight into the origin of the small post-edge shoulders near 6725 and 7320 eV observed in all of the Sm^II^ spectra. As shown by the dashed lines in Fig. 1 and 2, this post-edge feature corresponds to the peak maximum of Sm^III^. While the magnitude of this feature is invariant with temperature between 8 and 100 K, it shows significant intensity changes during our attempts to reproduce the data, *e.g.* from sample-to-sample. Hence, we attribute this feature to a small amount of Sm^III^ contamination, which likely arose from unwanted reactions with small amounts of O_2_ or H_2_O. Despite our best attempts, we were unsuccessful in obtaining completely pure Sm^II^ spectra; (1) analytes were shipped to the synchrotron cold and under vacuum, (2) XANES-samples were prepared at low temperature with rigorous exclusion of air and moisture immediately before the experiment, and (3) measurements were obtained rapidly (low temperature, under vacuum) using an unfocused beam. While it is difficult to identify what caused this contamination, the decomposition rate from X-ray radiolysis under our experimental conditions is slow. For example, when samples are cooled under vacuum (8 to 100 K; 10^–7^ Torr), the Sm^II^ spectra are unchanged after 3 hours of exposure to X-rays using an unfocused beam on SSRL's beam line 11-2. These results suggest that the Sm^III^ species is not being generated during the XANES data acquisition. However, we identified under different experimental conditions – using a focused beam at room temperature under an argon atmosphere on SSRL's beam line 6-2 – that complete conversion of Sm^II^(C_5_H_4_SiMe_3_)_3_^1–^ to Sm^III^ occurred in less than 10 seconds.

### Tm and Yb L_3,2_-edge XANES

**Fig. 3:**
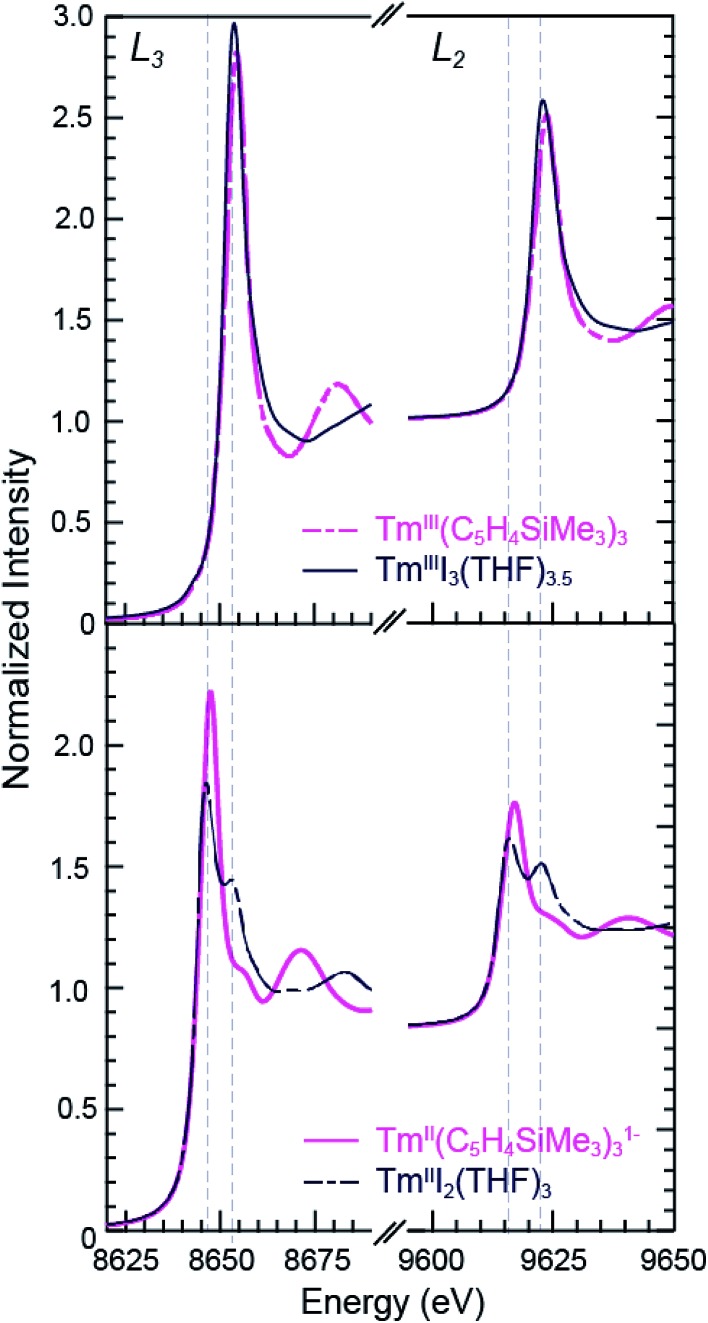
The background-subtracted and normalized Tm L_3,2_-edge XANES spectra obtained from Tm^III^(C_5_H_4_SiMe_3_)_3_ (top, pink dashed trace), TmI_3_(THF)_3.5_ (top, black trace), [K(2.2.2-cryptand)][Tm^II^(C_5_H_4_SiMe_3_)_3_] (bottom, pink trace), and TmI_2_(THF)_3_ (bottom, black dashed trace).

The background-subtracted and normalized Tm L_3,2_-edge XANES spectra from +2 and +3 thulium compounds are shown in Fig. 3. As observed for the samarium compounds in Fig. 1 and 2, spectra from the [K(2.2.2-cryptand)][Tm^II^(C_5_H_4_SiMe_3_)_3_] and Tm^II^I_2_(THF)_3_ compounds display two main features. There are pronounced peaks near 8645 eV (L_3_) and 9615 eV (L_2_) and higher energy post-edge shoulders at approximately 8655 eV and 9625 eV. Comparisons with +3 thulium compounds – namely, Tm^III^(C_5_H_4_SiMe_3_)_3_ and Tm^III^I_3_(THF)_3.5_ – lead us to interpret the Tm^II^ spectra in analogy to the Sm^II^ results described above. For instance, the large edge-features for Tm^II^(C_5_H_4_SiMe_3_)_3_^1–^ and Tm^II^I_2_(THF)_3_ are about 7 eV lower in energy than the edge features from Tm^III^(C_5_H_4_SiMe_3_)_3_ and Tm^III^I_3_(THF)_3.5_, Table 1. The spectral shapes and the trend toward lower energy for the Ln^II^ L_3_-edges from Ln(C_5_H_4_SiMe_3_)_3_^*x*–^ (Ln = Tm, Sm; *x* = 0, 1) are consistent with models of the data generated using quantum chemical *ab initio* FEFF9.6 code based on the multiple scattering theory (see Fig. S1 and S2†).^14^

As observed in the Sm^II^ L_3,2_-edge XANES experiments, the Tm^II^ spectra contain post-edge shoulders associated with small amounts of +3 thulium contamination. Variable temperature XANES experiments conducted between 8 and 100 K on these thulium compounds using a small excitation beam (1 × 1 mm) that was rastered across the sample show small variations in peak intensities. However, because the changes are not reversible and not reproducible, we attribute the slight variances to sample decomposition. Nevertheless, the compounds seem quite stable to X-ray radiation damage on the XANES experimental time scale (10 s to 1.5 h) under our experimental conditions; low temperature (8–100 K), under vacuum (10^–7^ Torr), and in an unfocused beam on SSRL's beam line 11-2.

**Fig. 4:**
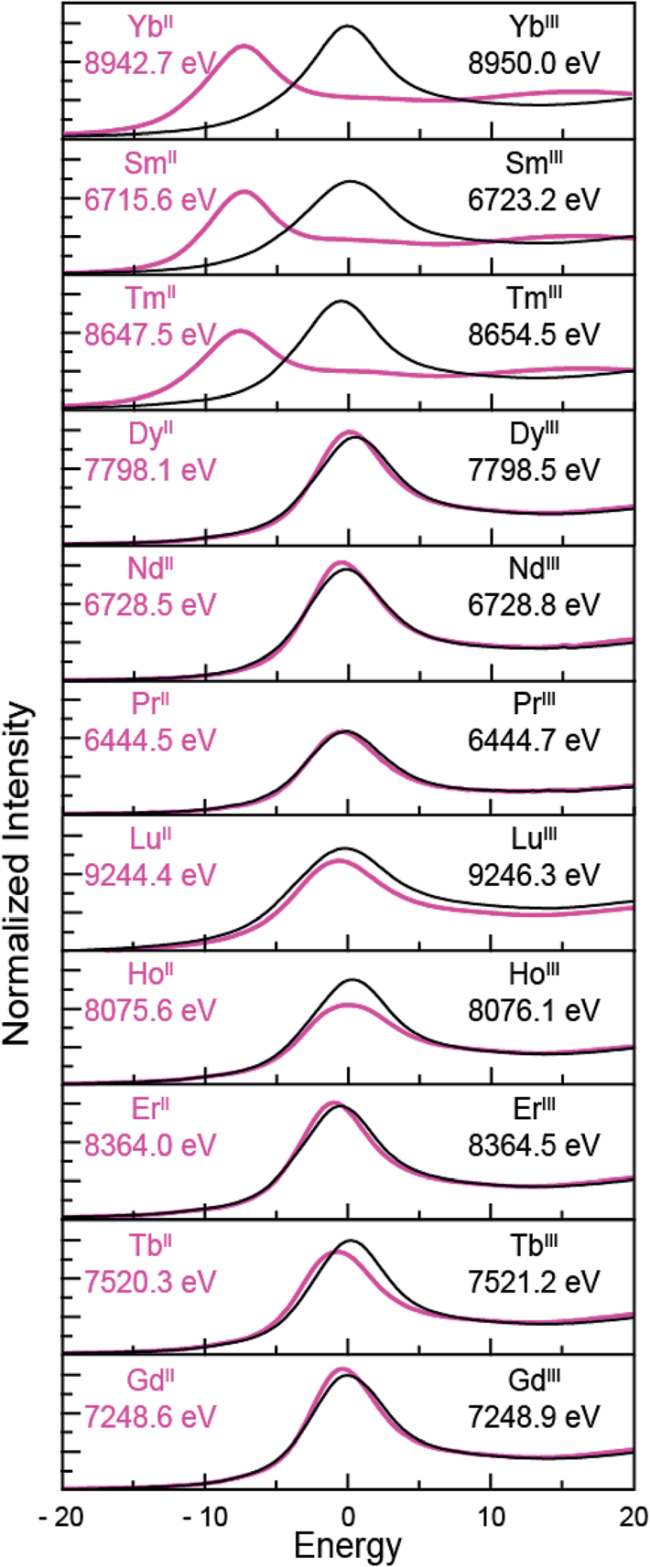
The background-subtracted and normalized L-edge XANES spectra obtained from Ln^III^(C_5_H_4_SiMe_3_)_3_ (black traces) and [K(2.2.2-cryptand)][Ln^II^(C_5_H_4_SiMe_3_)_3_] (pink traces) for Ln = Yb, Sm, Tm, Dy, Nd, Pr, Lu, Ho, Er, Tb and Gd. All spectra were collected at the Ln L_3_-edge except Nd and Pr, which were collected at the L_2_-edge. Peak maxima are shown in each pane. The spectra have been ordered from top to bottom based on increasing general reduction potentials.^4,16^

Despite minor Ln^III^ contamination in the Sm^II^ and Tm^II^ spectra, these results provide confidence and credibility in our abilities to manipulate extremely air and moisture sensitive organometallic complexes at the SSRL synchrotron facility. We remind the reader of the sensitivity of the Ln^III^(C_5_H_4_SiMe_3_)_3_ compounds to hydrolysis, the highly reducing nature of Sm^II^ and Tm^II^ (which have standard reduction potentials of –1.5 and –2.3 V *versus* SHE),^4^ and of the light sensitivity of Tm^III^I_3_(THF)_3.5_. As noted previously,^12,15^ the consistent 7–8 eV shift between Ln^II^ and Ln^III^ containing compounds highlights the utility of overcoming these sample handling challenges for characterizing Tm^II^ 4f^13^ 5d^0^ *versus* Tm^III^ 4f^12^ 5d^0^ electronic configurations using L_3,2_-edge XANES spectroscopy. Note that while not explicitly described here in detail, Fig. 4 shows that similar results were observed for ytterbium, whose spectrum, also displayed a peak maxima shift of ∼7 eV upon moving from Yb^II^ (4f^14^ 5d^0^) to Yb^III^ (4f^13^ 5d^0^).

### Ln^II^(C_5_H_4_SiMe_3_)_3_^1–^ Ln L_3,2_-edge (Ln = Pr, Nd, Gd, Tb, Dy, Ho, and Er) XANES

The samarium, thulium, and ytterbium L_3,2_-edge measurements described above provide an experimental basis for using XANES spectroscopy to evaluate the recently discovered Ln^II^(C_5_H_4_SiMe_3_)_3_^1–^ (Ln = Pr, Nd, Gd, Tb, Dy, Ho, and Er) compounds.^1a,1b,8^ Fig. 4 compares the background-subtracted and normalized Ln L_3_- or L_2_-edge XANES spectra from [K(2.2.2-cryptand)][Ln^II^(C_5_H_4_SiMe_3_)_3_] with Ln^III^(C_5_H_4_SiMe_3_)_3_. In this figure, the spectra are ordered from top to bottom as a function of increasing standard reduction potential, as determined by Morss and Mikheev.^4,16^ These data display rising-edge features similar to the samarium and thulium spectra described above. However, in stark contrast to the samarium, thulium, and ytterbium spectra, the L-edge peak maxima from the other Ln^II^(C_5_H_4_SiMe_3_)_3_^1–^ anions are quite similar in energy to the neutral Ln^III^(C_5_H_4_SiMe_3_)_3_ compounds. As shown in Fig. 4 and Table 1, small shifts in L_3_-edge inflection points are observed for the other Ln(C_5_H_4_SiMe_3_)_3_^*x*–^ (*x* = 0, 1) compounds, ranging from 0.2 to 1.0 eV.

**Fig. 5:**
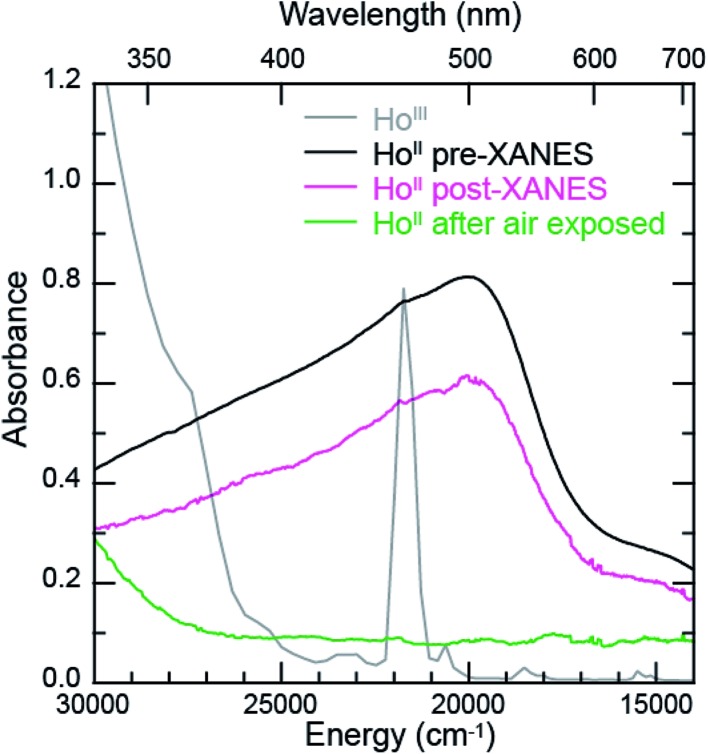
The background-subtracted UV-vis spectra obtained from Ho^III^(C_5_H_4_SiMe_3_)_3_ (grey trace) and [K(2.2.2-cryptand)][Ho^II^(C_5_H_4_SiMe_3_)_3_]. Data from Ho^II^(C_5_H_4_SiMe_3_)_3_^1–^ were collected (1^st^) before XANES analysis (black trace), (2^nd^) after XANES analysis (pink trace), and (3^rd^) after XANES analysis and exposure to air (green trace).

To evaluate the likelihood that the spectra obtained from Ln^II^(C_5_H_4_SiMe_3_)_3_^1–^ (Ln = Pr, Nd, Gd, Tb, Dy, Ho, and Er) compounds were indeed correct, a series of control experiments were conducted. Herein we limit the discussion explicitly to the Ho^II^/Ho^III^ case. The first control experiment involved analyzing the Ho^II^ and Ho^III^ samples by electronic absorption spectroscopy before and after the Ho L_3,2_-edge XANES experiment. Because the Ho^II^(C_5_H_4_SiMe_3_)_3_^1–^ UV-vis spectrum is distinct from the Ho^III^(C_5_H_4_SiMe_3_)_3_ precursor, electronic absorption spectroscopy provides a robust method for confirming the presence of Ho^II^(C_5_H_4_SiMe_3_)_3_^1–^ during the XANES experiment. First, an aliquot of Ho^II^(C_5_H_4_SiMe_3_)_3_^1–^ was characterized by UV-visible spectroscopy (black trace, Fig. 5; pre-XANES). The spectrum showed the characteristic and broad charge transfer band associated with Ho^II^(C_5_H_4_SiMe_3_)_3_^1–^. Moreover, no detectible Ho^III^ was observed. For comparison, the spectrum from Ho^III^(C_5_H_4_SiMe_3_)_3_ is shown as a gray trace. A second aliquot of the Ho^II^(C_5_H_4_SiMe_3_)_3_^1–^ was diluted in BN and the Ho L_3,2_-edge XANES experiment was conducted. Subsequently, the sample – Ho^II^(C_5_H_4_SiMe_3_)_3_^1–^ and BN – was transferred to a Teflon sealable quartz cuvette and the mixture was again characterized by UV-visible spectroscopy (pink trace, post-XANES). Unfortunately, because of constraints associated with the XANES holder, this transfer was not quantitative and the overall amount of Ho^II^(C_5_H_4_SiMe_3_)_3_^1–^ in the cuvette was unknown. A 20% loss during the transfer is possible. Hence, the intensities in the pre-XANES spectrum cannot be directly compared with those from the post-XANES spectrum. Additionally, the BN in the post-XANES spectrum is insoluble and artificially increases the overall UV-visible baseline due to scattering effects. For data comparison, the post-XANES spectrum was background-subtracted to place overall peak heights on the same approximate absorbance scale. Regardless, this experiment unambiguously demonstrates that no detectable amount of Ho^III^(C_5_H_4_SiMe_3_)_3_ was observed before or after the synchrotron experiment. One cannot rule out the possibility of insoluble Ho^III^ contaminates. For example, exposing a Teflon sealable cuvette containing the Ho^II^(C_5_H_4_SiMe_3_)_3_^1–^ post-XANES samples to air for 2 s caused an immediate loss of Ho^II^ signal and no ingrowth of Ho^III^ 4f → 4f transitions. However, when one considers loss of sample during the transfer from the XANES holder to the cuvette, this control experiment suggests that after the Ho L_3,2_-edge experiment >80% of the sample was in the form of Ho^II^(C_5_H_4_SiMe_3_)_3_^1–^.

**Fig. 6:**
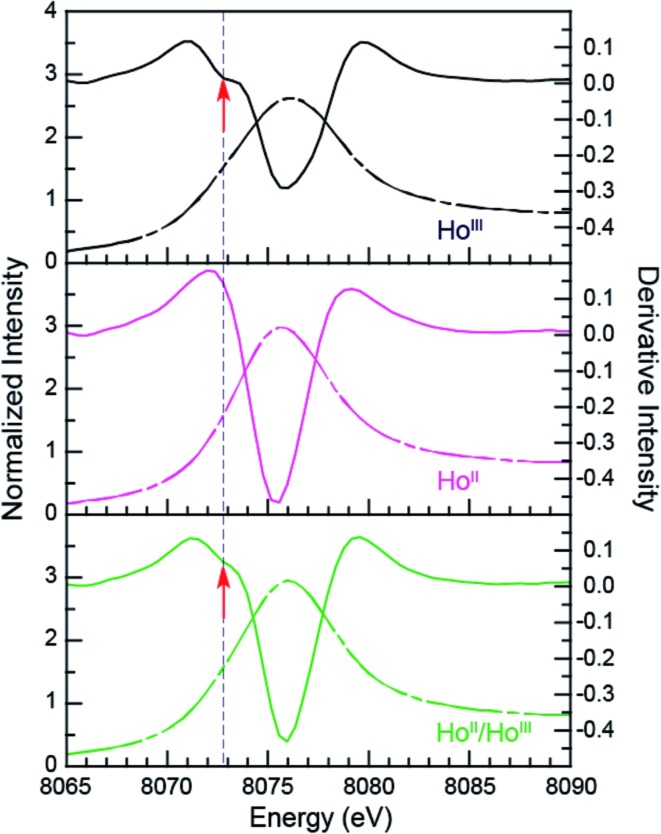
The background-subtracted and normalized Ho L_3_-edge XANES measurements obtained from Ho^III^(C_5_H_4_SiMe_3_)_3_ (black dashed trace), [K(2.2.2-cryptand)][Ho^II^(C_5_H_4_SiMe_3_)_3_] (pink dashed trace) complexes, and a mixture of Ho^III^ and Ho^II^ samples (green dashed trace). Second derivatives of the data are provides as solid traces. Note the pre-edge features (labeled with a red arrow) that are manifested as a minimum in the 2^nd^ derivative.

Additional support that the Ho L_3,2_-edge XANES spectra obtained from Ho^II^(C_5_H_4_SiMe_3_)_3_^1–^ was representative of the Ho^II^ organometallic was gleaned from a series of X-ray absorption decomposition experiments. For example, exposing Ho^II^(C_5_H_4_SiMe_3_)_3_^1–^, whose absorption peak is at 8075.6 eV, after XANES analysis to air caused the peak position to shift by 0.5 eV to 8076.0 eV, matching the 8076.1 eV peak in Ho^III^(C_5_H_4_SiMe_3_)_3_. Analysis of the second derivative of the Ln^II^ *versus* Ln^III^ data additionally revealed a spectral diagnostic for the unconventional Ln^II^(C_5_H_4_SiMe_3_)_3_^1–^ (Fig. S4†) compounds. For example, all of the +3 Ln^III^(C_5_H_4_SiMe_3_)_3_ precursors contain a minimum in the second derivative approximately 2 eV lower in energy than the corresponding absorption peak. For Sm, Tm, and Yb, this feature is also persists after reduction to the Ln^II^(C_5_H_4_SiMe_3_)_3_^1–^ complex. However, reduction to form unconventional divalents, Ln = Gd, Tb, Dy, Ho, Er, and Lu, caused the pre-edge features to disappear from the L_3_-edges XANES spectra. This observation is documented by the 2^nd^ derivative plots shown in Fig. 6 for Ho(C_5_H_4_SiMe_3_)_3_ *^x^* ^–^ (*x* = 1, 0) (see ESI† for the other L_3_-edge 2^nd^ derivative spectra). We remind the reader that a minimum in the 2^nd^ derivative indicates the presence of a peak in the XANES data. Fig. 6 shows the pre-edge peak at 8073.0 eV for Ho^III^(C_5_H_4_SiMe_3_)_3_. If the transition corresponds to a Ln 2p → 5d excitation, 5d-orbital population in Ln^II^(C_5_H_4_SiMe_3_)_3_^1–^ would shift this feature higher in energy (owing to electron pairing energy) and make it more difficult to resolve. Consistent with this proposition, for Sm, Tm, and Yb analytes – which have 4f^*n*^ 5d^0^ (for +3 metals) and 4f^*n*+1^ 5d^0^ (for +2 metals) electronic configurations with empty 5d orbitals (for both +3 and +2 metals) – pre-edge features were observed in both the +3 and +2 spectra. Regardless of its identity, this pre-edge feature is unexpectedly sensitive to the amount of Ln^III^ present in the Ln^II^ sample, as demonstrated by the Ho L_3_-edge XANES measurement made on a 1 : 1 mixture of Ho^III^(C_5_H_4_SiMe_3_)_3_ and Ho^II^(C_5_H_4_SiMe_3_)_3_^1–^, Fig. 6, which showed the pre-edge feature had a lower intensity than the pure Ho^III^ starting material. The absence of the extra feature in the Ln^II^(C_5_H_4_SiMe_3_)_3_^1–^ L_3_-edge XANES spectra provides a fortuitous alternative fingerprint for the Ln^II^ compounds with 4f^*n*^ 5d^1^ electronic configurations. This is especially valuable when one considers that L_3_/L_2_ absorption peak area comparisons and branching ratio analyses were inconclusive (Table S1†), even for the Sm, Tm, and Yb analytes.

### M^II^(C_5_H_4_SiMe_3_)_3_^1–^ K- and L_3,2_-edge XANES (M = Y, Lu)

**Fig. 7:**
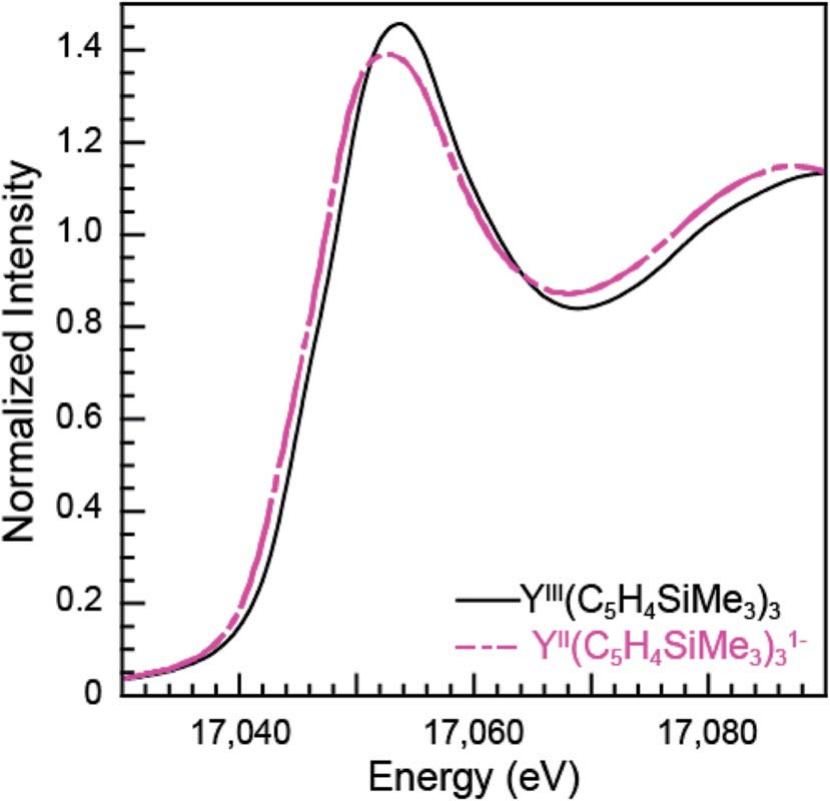
The background-subtracted and normalized Y K-edge XANES measurements obtained from Y^III^(C_5_H_4_SiMe_3_)_3_ (black trace) and [K(2.2.2-cryptand)][Y^II^(C_5_H_4_SiMe_3_)_3_] (pink dashed trace) complexes.

The subtle rising edge energy shifts between Ln^II^(C_5_H_4_SiMe_3_)_3_^1–^ and Ln^III^(C_5_H_4_SiMe_3_)_3_ are reminiscent of those accompanying changes in oxidation state for transition metals (K- and L-edges),^17,18^ not lanthanides. For example, changes in d-orbital occupancy only shift the K- and L-edges for transition metals by a few electron volts, which pales in comparison to the 7 eV shifts that accompany oxidation state changes in 4f-element chemistry. For example, the Y K-edge XANES data from Y^II^(C_5_H_4_SiMe_3_)_3_^1–^ and Y^III^(C_5_H_4_SiMe_3_)_3_ show a 1.4 eV inflection point shift (Fig. 7, Table 1), which is consistent with the computational results generated using quantum chemical *ab initio* FEFF9.6 code based on the multiple scattering theory (see Fig. S3†).^14^ Hence, both experiment and theory indicate that Y^III^(C_5_H_4_SiMe_3_)_3_ has a 4d^0^ electronic configuration and Y^II^(C_5_H_4_SiMe_3_)_3_^1–^ a 4d^1^ configuration. These Y K-edge XANES results agree with the previous analyses of Y^II^(C_5_H_4_SiMe_3_)_3_^1–^ (UV-vis, EPR, structural metrics)^1*c*^ and – to the best of our knowledge – represent the first Y K-edge XANES spectrum of a molecule containing Y^II^. Also consider data from the Lu(C_5_H_4_SiMe_3_)_3_^*x*–^ (*x* = 0, 1) pair. Lutetium in the +3 oxidation state has a full 4f-shell. Hence reduction of Lu^III^(C_5_H_4_SiMe_3_)_3_, with a 4f^14^ 5d^0^ electron configuration, has to generate a 4f^14^ 5d^1^ configuration in Lu^II^(C_5_H_4_SiMe_3_)_3_^1–^. Consistent with 5d-orbital occupation, the peak maxima difference between Lu^III^ and Lu^II^ in the Lu L_3,2_-edge XANES was small, measured at 1.9 eV.

Taken in the context of these Y(C_5_H_4_SiMe_3_)_3_^*x*–^ and Lu(C_5_H_4_SiMe_3_)_3_^*x*–^ (*x* = 0, 1) XANES measurements – alongside (1^st^) the experiments we conducted showing our XANES samples contained only marginal quantities of Ln^III^ decomposition products, and (2^nd^) previously reported UV-vis data, structural metrics, previous computational results – the most plausible interpretations of these Ln L_3_-edge XANES data (Fig. 4) is that reduction of Ln^III^(C_5_H_4_SiMe_3_)_3_ to form an unconventional Ln^II^(C_5_H_4_SiMe_3_)_3_^1–^ compound resulted in addition of an electron into a highly shielded 5d-orbital to generate a 4f^n^ 5d^1^ electronic configuration, not 4f^*n*+1^ 5d^0^. Although we anticipate that the spectra in Fig. 4 contain some Ln^III^ contamination – in analogy to the Sm^II^ and Tm^II^ spectra in Fig. 1 to 3 – the computational results below provide even more support for the alternative electronic configuration.

### Electronic structure calculations

To better understand the origin for the spectroscopic differences between Ln^III^(C_5_H_4_SiMe_3_)_3_ *versus* Ln^II^(C_5_H_4_SiMe_3_)_3_^1–^, electronic structure calculations were conducted on a subset of Ln(C_5_H_4_SiMe_3_)_3_^*x*–^ (Ln = Sm, Ho; *x* = 0, 1) complexes. This analysis compares Sm^II^(C_5_H_4_SiMe_3_)_3_^1–^, which is unambiguously +2, with Ho^II^(C_5_H_4_SiMe_3_)_3_^1–^, where the electronic configuration is ambiguous. Calculations for the Ln^II^(C_5_H_4_SiMe_3_)_3_^1–^ compounds were restricted to just Sm and Ho, as a follow-on manuscript will compare theoretical results from the other Ln^II^ compounds with other +2 lanthanide and actinide species. Initially, DFT/PBE calculations were conducted to optimize the geometric structures of Ln(C_5_H_4_SiMe_3_)_3_^*x*–^ (*x* = 1, 0), see Table 2 for a comparison of experimental and calculated distances and Table S2 (ESI†) for the coordinates. The computational results reveal a ground-state 4f^5^ 5d^0^ electronic configuration (sextet state) for Sm^III^(C_5_H_4_SiMe_3_)_3_ and a 4f^6^ 5d^0^ (septet state) configuration for Sm^II^(C_5_H_4_SiMe_3_)_3_^1–^. Because of the near-degeneracy of 4f-orbitals and the accompanying marginal participation in metal–ligand covalent bonding,^11*e*^ varying 4f-occupations of the ground-state spin multiplicity has little effects on the geometric structures and spectra. The average 2.513 Å Sm^III^–C_centroid_ distance is calculated to be 0.092 Å shorter than the 2.605 Å Sm^II^–C_centroid_ distance. This difference is consistent with the differences in Sm^III^ *versus* Sm^II^ ionic radii^19^ and changes in electrostatic interactions between Sm^III^ *versus* Sm^II^ with C_5_H_4_SiMe_3_^1–^ anions. These calculated distances compare well with experimental results^8*a*^ and are within the typical error of 2% observed for GGA functionals.

**Table 2:** The DFT/PBE calculated and experimental Ln–C_centroid_ (Cnt) distances (Å) from Ln^III^(C_5_H_4_SiMe_3_)_3_ and Ln^II^(C_5_H_4_SiMe_3_)_3_^1–^ (Ln = Sm, Ho). Structural metrics from Ho^II^(C_5_H_4_SiMe_3_)_3_^1–^ with 4f^10^ 5d^1^ *versus* 4f^11^ 5d^0^ electronic configurations were also compared

Sm(C_5_H_4_SiMe_3_)_3_^*x*–^ (*x* = 0, 1)						
	Sm^III^, 4f^5^ 5d^0^		Sm^II^, 4f^6^ 5d^0^		Δ(Sm^II^–Sm^III^)	
	PBE	Exp^8*a*^	PBE	Exp^8*a*^	PBE	Exp^8*a*^
Sm–Cnt1	2.508	2.459	2.610	2.603	0.102	0.144
Sm–Cnt2	2.512	2.459	2.595	2.607	0.083	0.148
Sm–Cnt3	2.519	2.464	2.609	2.615	0.090	0.151
Avg(Sm–Cnt)	2.513	2.461	2.605	2.608	0.092	0.147

Ho(C_5_H_4_SiMe_3_)_3_^*x*–^ (*x* = 0, 1)							
	Ho^III^, 4f^10^ 5d^0^		Ho^II^, 4f^10^ 5d^1^		Ho^II^, 4f^11^ 5d^0^	Δ[Ho^II^ (4f^10^ 5d^1^)–Ho^III^]	
	PBE	Exp^1*b*^	PBE	Exp^1*b*^	PBE	PBE	Exp^1*b*^
Ho–Cnt1	2.438	2.391	2.477	2.417	2.536	0.039	0.026
Ho–Cnt2	2.441	2.393	2.461	2.420	2.509	0.020	0.027
Ho–Cnt3	2.448	2.398	2.481	2.432	2.517	0.033	0.034
Avg(Ho–Cnt)	2.442	2.394	2.473	2.423	2.521	0.031	0.029

Consistent with previous hybrid DFT calculations that employed no less than 25% Hartree–Fock (HF) exchange,^1*b*^ our calculations show the ground-state electronic structure of Ho^III^(C_5_H_4_SiMe_3_)_3_ is 4f^10^ 5d^0^ (quintet state), whereas Ho^II^(C_5_H_4_SiMe_3_)_3_^1–^ has a 4f^10^ 5d^1^ configuration (sextet state). For example, calculations with the BHandHLYP functional show the 4f^10^ 5d^1^ electronic configuration is 27 kcal mol^–1^ more stable than the alternative 4f^11^ 5d^0^ configuration (quartet state). In contrast, calculations with functionals that included less HF exchange (PBE, BLYP, and B3LYP) incorrectly predict the alternative Ho^II^ 4f^11^ 5d^0^ configuration as the ground-state (see details in Tables S2 and S3 of the ESI†).^1b,1c,8a,8d^ That is to say, GGA and hybrid functionals with lower HF exchange percentages fail to give the correct Ho^II^(C_5_H_4_SiMe_3_)_3_^1–^ spin state, which is likely attributable to the delocalization error.^20,21^ Many reports have described how increasing HF exchange improves the calculated energetics by DFT-based methods such as excitation energy,^22^ thermochemical kinetics,^23^ reaction barriers,^24^ and electron detachment energy.^25^ Consistently, our DFT/PBE calculated Ho^III^ (4f^10^ 5d^0^)–C_centroid_ and Ho^II^ (4f^10^ 5d^1^)–C_centroid_ distances are in excellent agreement with experimental values (Table 2), while the Ho^II^ (4f^11^ 5d^0^)–C_centroid_ distances are longer than the experimental results by ∼0.1 Å.^1b,1c^ These results provide confidence in assigning Ho^II^ as having a 4f^10^ 5d^1^ electronic configuration. We refer the interested reader to the experimental section for details of the electronic structure calculation.

**Fig. 8:**
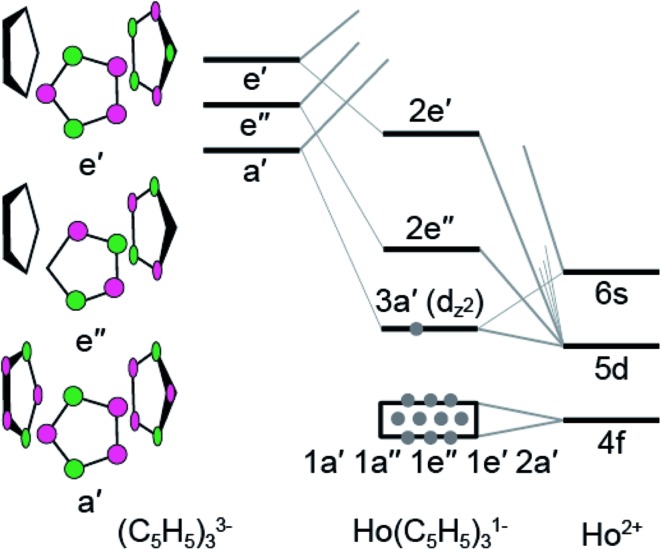
A qualitative molecular diagram showing molecular orbital interactions in *C*_3h_-symmetry for Ho^II^(C_5_H_5_)_3_^1–^.

To better understand the unusual electronic configuration of Ho^II^(C_5_H_4_SiMe_3_)_3_^1–^, we found it instructive to interpret the DFT calculations using traditional molecular orbital descriptions derived from group theory considerations of M(C_5_H_5_)_3_ in *C*_3h_-symmetry. Hence, a qualitative MO level diagram for the *C*_3h_–Ho^II^(C_5_H_5_)_3_^1–^ anion is provided in Fig. 8. As the molecular orbital interactions associated with Ln^III^(C_5_R_5_)_3_ (R = H or alkyl) have been the subject of numerous theoretical and spectroscopic studies,^26^ this discussion is confined to those orbitals most relevant to the Sm and Ho L_3,2_-edge XANES measurements. In contrast to previous theoretical results for M^III^(C_5_H_5_)_3_ in *D*_3h_- or *C*_3v_-symmetry,^26b,c,d,g,h,i,j^ we find it more appropriate to describe the MO-interaction using *C*_3h_-symmetry, as this designation more closely mimics data from the crystal structure of Ho^II^(C_5_H_4_SiMe_3_)_3_^1–^.

In the *C*_3h_-point group, symmetry allowed mixing between the metal 5d- and cyclopentadienyl π-orbitals – perpendicular to the ring planes – generates bonding interactions of *a*′, *e*′, and *e*′′ symmetries, which were σ- π- and δ-bonding with respect to the metal–cyclopentadienyl centroid axes, Fig. 8. Superimposed on this molecular orbital picture, and at lower energy, are Ln–(C_5_H_5_) σ-, π- and δ-bonding orbitals of *a*′, *a*′′, *e*′, and *e*′′ symmetries that originate from mixing between the 4f-orbitals and cyclopentadienyl π-orbitals. In general, the Ln(C_5_H_4_SiMe_3_)_3_^*x*–^ (*x* = 0, 1) compounds exhibit little 4f- and cyclopentadienyl orbital mixing, such that the seven primarily 4f-orbitals span a narrow energy range. In contrast, substantial mixing occurs between the Ln 5d- and cyclopentadienyl π-orbitals, with the exception of the 5d-orbital of 3*a*′ symmetry (*d*_z2_). Consistent with previous reports,^1*b*^ metal–cyclopentadienyl mixing is inhibited in this 3*a*′ orbital by poor spatial overlap. Hence, the 3*a*′ orbital is primarily composed of 5d- and 6s-character and best described as a non-bonding 5d-orbital. For Sm^III^(C_5_H_4_SiMe_3_)_3_ (4f^5^ 5d^0^), Sm^II^(C_5_H_4_SiMe_3_)_3_^1–^ (4f^6^ 5d^0^), and Ho^III^(C_5_H_4_SiMe_3_)_3_ (4f^10^ 5d^0^), the 3*a*′ orbital is empty. As testament, the BHandHLYP calculations at PBE optimized ground-state geometries show the Mulliken net spin densities^27^ to be almost exclusively distributed on 4f-orbitals (Table 3). Meanwhile, for the Ho^II^(C_5_H_4_SiMe_3_)_3_^1–^ anion (4f^10^ 5d^1^), significant 6s- and 5d-spin density distribution associated with the highest alpha spin occupied orbital indicates that the 3*a*′ orbital is singly occupied. A summary of the spin multiplicity results is provided in Table 3. The differences between the 4f^*n*+1^ 5d^0^ *versus* 4f^*n*^ 5d^1^ electronic configurations of the Ln^II^ ions is observed to influence the metal–cyclopentadienyl bond distances and, as discussed below, is found to significantly impact the Ln L_3_-edge XANES spectrum.

**Table 3:** The spin multiplicity (2S + 1), total S^2^, Mulliken net spin density for lanthanide atomic electron valence orbitals (s, d, f) calculated for Ln(C_5_H_4_SiMe_3_)_3_^*x*–^ (Ln = Sm, Ho; *x* = 0, 1) using DFT/BHandHLYP

Compound		2S + 1	S^2^	Spin	s	d	f
Sm^III^(C_5_H_4_SiMe_3_)_3_	4f^5^ 5d^0^	6	8.77	5.14	0.01	0.09	5.03
Sm^II^(C_5_H_4_SiMe_3_)_3_^1–^	4f^6^ 5d^0^	7	12.01	6.04	0.01	0.06	5.96
Ho^III^(C_5_H_4_SiMe_3_)_3_	4f^10^ 5d^0^	5	6.00	4.04	0.00	0.04	3.97
Ho^II^(C_5_H_4_SiMe_3_)_3_^1–^	4f^10^ 5d^1^	6	8.76	4.86	0.22	0.62	3.98
Ho^II^(C_5_H_4_SiMe_3_)_3_^1–^	4f^11^ 5d^0^	4	3.76	3.02	0.00	0.01	3.01

To support the results from the ground-state DFT calculations, CASPT2/CASSCF calculations were performed on the ground-states and low excited-states of simplified Ln(C_5_H_5_)_3_^*x*–^ (Ln = Sm, Ho; *x* = 0, 1) complexes. The DFT/PBE optimized geometries of Ln(C_5_H_4_SiMe_3_)_3_^*x*–^ were used; however, to reduce the computational cost SiMe_3_ substituents were replaced with protons having C–H bond lengths of 1.088 Å. Two possibilities were investigated for Ho^II^(C_5_H_5_)_3_^1–^. The first was associated with the calculated structure of Ho^II^(C_5_H_4_SiMe_3_)_3_^1–^ with a 4f^10^ 5d^1^ ground-state electronic configuration. The second investigated Ho^II^(C_5_H_5_)_3_^1–^ geometry was based on the calculated 4f^11^ 5d^0^ Ho^II^(C_5_H_4_SiMe_3_)_3_^1–^ structure. Although efforts were made to include all the seven 4f and five 5d orbitals into the active space, the converged CASSCF results for Sm(C_5_H_5_)_3_^*x*–^ (*x* = 0, 1) showed that the five 5d-orbitals were not correlated and removed from the active space. Meanwhile for Ho(C_5_H_5_)_3_^*x*–^ (*x* = 0, 1), only the 5d_z2_-orbital remained in the active space. Hence, the active space calculations were adjusted to include all seven 4f-orbitals for Sm(C_5_H_5_)_3_^*x*–^ (*x* = 0, 1) and an additionally 5d_z2_-orbital for Ho(C_5_H_5_)_3_^*x*–^ (*x* = 0, 1). The results generated a complete active space of 6-electrons with 7-orbitals for Sm^II^(C_5_H_5_)_3_^1–^, 5-electrons and 7-orbitals for Sm^III^(C_5_H_5_)_3_, 11-electrons and 8-orbitals for Ho^II^(C_5_H_5_)_3_^1–^, and 10-electrons with 8-orbitals for Ho^III^(C_5_H_5_)_3_.

Although subtle differences were observed, the ground-state electronic structure results from the CASPT2/CASSCF calculations are similar to those obtained by DFT (Table 4). The “core-like” and nearly degenerated 4f-orbitals resulted in different 4f-occupations with nearly the same energies. The CASPT2/CASSCF results show that Sm^III^(C_5_H_5_)_3_ has ground sextet state of 4f^5^ configurations and that Sm^II^(C_5_H_5_)_3_^1–^ has ground septet state of 4f^6^ configuration, which are the same as DFT results. In the holmium case, Ho^III^(C_5_H_5_)_3_ has ground quintet state of 4f^10^ 5d^0^. For Ho^II^, both geometries showed a sextet with 4f^10^ 5d^1^ configurations. These Ho^II^ and Ho^III^ results were identical to the DFT calculations. Hence, in terms of evaluating ground-state electronic structures for the Ln(C_5_H_5_)_3_^*x*–^ (*x* = 0, 1), the CASPT2/CASSCF results are in excellent agreement with the reported DFT results from Ln(C_5_H_4_SiMe_3_)_3_^*x*–^ (*x* = 0, 1).

**Table 4:** Ground-states configurations from Ln(C_5_H_5_)_3_^*x*–^ (Ln = Sm, Ho; *x* = 0, 1) complexes from CASPT2/CASSCF calculations.^*a*^ Geometries relied on the DFT/PBE optimized geometries of Ln(C_5_H_4_SiMe_3_)_3_^*x*–^. However, for Ho^II^(C_5_H_5_)_3_^1–^ two geometries were investigated that were derived from the calculated Ho^II^(C_5_H_3_SiMe_3_)_3_^1–^ structures with either 4f^10^ 5d^1^ or 4f^11^ 5d^0^ electronic configurations

Ground-state	Configurations
**Sm** ^**II**^ **(C**_**5**_ **H**_**5**_ **)**_**3**_^**1–**^	
X^7^A	100%(1a^1^2a^1^3a^1^4a^1^5a^1^6a^1^7a^0^)

**Sm** ^**III**^ **(C**_**5**_ **H**_**5**_ **)**_**3**_	
X^6^A	58%(1a^1^2a^1^3a^1^4a^1^5a^1^6a^0^7a^0^) + 41%(1a^1^2a^1^3a^1^4a^0^5a^0^6a^1^7a^1^)

**Ho(C**_**5**_ **H**_**5**_ **)**_**3**_^**1–**^ **; geometry from Ho** ^**II**^ **(C**_**5**_ **H**_**4**_ **SiMe**_**3**_ **)**_**3**_^**1–**^ **(4f** ^**10**^ **5d** ^**1**^ **)** ^*b*^	
X^6^A	71%(1a^2^2a^2^3a^1^4a^2^5a^1^6a^1^7a^1^8a^1^) + 21%(1a^2^2a^1^3a^2^4a^1^5a^2^6a^1^7a^1^8a^1^) + 7%(1a^1^2a^2^3a^2^4a^1^5a^1^6a^2^7a^1^8a^1^)

**Ho(C**_**5**_ **H**_**5**_ **)**_**3**_^**1–**^ **; geometry from Ho** ^**II**^ **(C**_**5**_ **H**_**4**_ **SiMe**_**3**_ **)**_**3**_^**1–**^ **(4f** ^**11**^ **5d** ^**0**^ **)** ^*b*^	
X^6^A	70%(1a^2^2a^2^3a^1^4a^2^5a^1^6a^1^7a^1^8a^1^) + 21%(1a^2^2a^1^3a^2^4a^1^5a^2^6a^1^7a^1^8a^1^) + 7%(1a^1^2a^2^3a^2^4a^1^5a^1^6a^2^7a^1^8a^1^)

**Ho(C**_**5**_ **H**_**5**_ **)**_**3**_	
X^5^A	65%(1a^2^2a^1^3a^2^4a^1^5a^2^6a^1^7a^1^8a^0^) + 20%(1a^2^2a^2^3a^1^4a^2^5a^1^6a^1^7a^1^8a^0^) + 5%(1a^1^2a^2^3a^2^4a^1^5a^1^6a^1^7a^2^8a^0^) + 2%(1a^2^2a^1^3a^1^4a^1^5a^2^6a^2^7a^1^8a^0^) + 1%(1a^2^2a^1^3a^1^4a^2^5a^2^6a^1^7a^1^8a^0^) + 1%(1a^2^2a^1^3a^2^4a^1^5a^1^6a^1^7a^2^8a^0^) + 1%(1a^2^2a^2^3a^2^4a^1^5a^1^6a^1^7a^1^8a^0^)

^*a*^1a-7a are 4f orbitals, and 8a is 5d orbital.

^*b*^Refer to the DFT/PBE calculated ground-state geometrics for Ho^II^(4f^10^5d^1^) and Ho^II^(4f^11^5d^0^), respectively, shown in Table 2.

### Spectral simulations

**Fig. 9:**
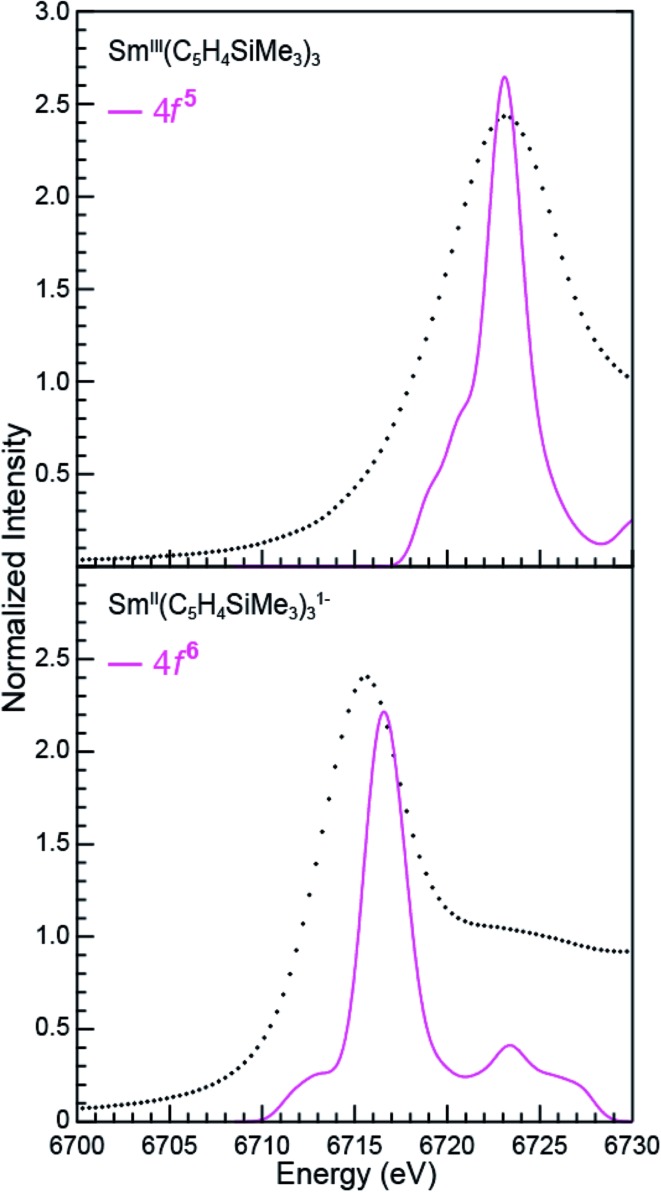
A comparison of the experimental (•) and transition dipole moment calculations (pink traces) for the Sm L_3_-edge XANES measurements obtained from Sm^III^(C_5_H_4_SiMe_3_)_3_ (top) and [K(2.2.2-cryptand)][Sm^II^(C_5_H_4_SiMe_3_)_3_] (bottom). The calculated spectra were shifted by a constant 241.49 eV, which aligned the Sm^III^(C_5_H_4_SiMe_3_)_3_ experimental and calculated edge peak.

**Fig. 10:**
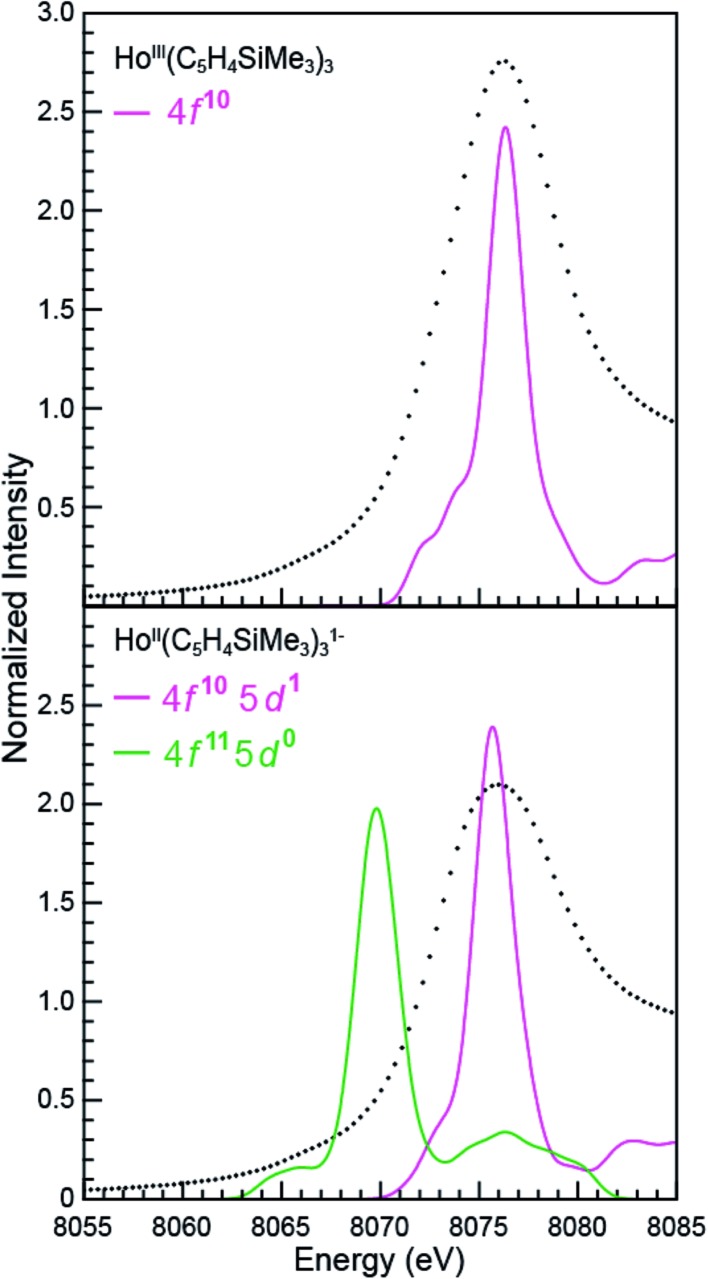
A comparison of the experimental (•) and transition dipole moment calculations (pink and green traces) for the Ho L_3_-edge XANES obtained from Ho^III^(C_5_H_4_SiMe_3_)_3_ (top) and [K(2.2.2-cryptand)][Ho^II^(C_5_H_4_SiMe_3_)_3_] (bottom). The calculated spectra were shifted by a constant 348.17 eV, which aligned the Ho^III^(C_5_H_4_SiMe_3_)_3_ experimental and calculated edge peak.

The open-shell Sm and Ho L_3_-edge XANES spectra from Ln(C_5_H_4_SiMe_3_)_3_^*x*–^ (Ln = Sm, Ho; *x* = 0, 1), were calculated using the transition dipole moment approach based on the Kohn–Sham ground-state molecular orbitals. Using this method the core excitation energies were calculated as the energy differences between occupied and virtual orbitals. Previous studies have demonstrated that this approach provides a sound basis for interpreting the experimental XANES spectra.^28^ BHandHLYP simulated Ln L_3_-edge XANES spectra from Ln(C_5_H_4_SiMe_3_)_3_^*x*–^ are compared with experimental results in Fig. 9 and 10. In these figures, the calculated spectra were shifted by a constant 241.49 eV (Sm) and 348.17 eV (Ho) to line up the Ln^III^(C_5_H_4_SiMe_3_)_3_ L_3_-edge peaks, which in turn accounts for omission of the atomic and extra-atomic relaxation associated with the core excitation, relativistic stabilization, and errors associated with the functionals.^29,30^ In the Ln^II^ cases, two options were explored, transitions that involved conventional electronic configurations, Ln^II^ 2p^6^…4f^*n*+1^ 5d^0^ → Ln^II^ 2p^5^…4f^*n*+1^ 5d^1^, and alternatives that involved 5d-orbital occupations, Ln^II^ 2p^6^…4f^*n*^ 5d^1^ → Ln^II^ 2p^5^…4f^1^ 5d^2^. The resulting near edge energies are summarized in Table 5 alongside analogous values acquired using PBE, BLYP, and B3LYP functionals.

**Table 5:** DFT calculated and experimental peak maximum for the Ln(C_5_H_4_SiMe_3_)_3_ *^x^* ^–^ (Ln = Sm, Ho; *x* = 0, 1) XANES spectra

		PBE	BLYP	B3LYP	BHandHLYP	Exp.
Sm^III^(C_5_H_4_SiMe_3_)_3_	4f^5^ 5d^0^	6873.4	6874.8	6910.7	6964.6	6723.2
Sm^II^(C_5_H_4_SiMe_3_)_3_^1–^	4f^6^ 5d^0^	6870.8	6872.2	6906.1	6958.1	6715.6
Δ[Sm^III^–Sm^II^]		2.6	2.6	4.6	6.5	7.6
Ho^III^(C_5_H_4_SiMe_3_)_3_	4f^10^ 5d^0^	8325.6	8327.1	8366.6	8424.5	8076.1
Ho^II^(C_5_H_4_SiMe_3_)_3_^1–^	4f^10^ 5d^1^	8325.8	8327.3	8366.2	8423.8	8075.6
Ho^II^(C_5_H_4_SiMe_3_)_3_^1–^	4f^11^ 5d^0^	8322.6	8324.1	8361.1	8418.0	—
Δ[Ho^III^–Ho^II^ (4f^10^ 5d^1^)]		–0.2	–0.2	0.4	0.7	0.5
Δ[Ho^III^–Ho^II^ (4f^11^ 5d^0^)]		3.0	3.0	5.5	6.5	—

The theoretical analyses reveal the primary contributions to the Ln L_3_-edge XANES spectra are electric dipole allowed excitations from Ln 2p-orbitals to unoccupied states that contain metal d-character. Of the functionals explored, the L_3_-edge energy differences calculated using BHandHLYP were in best agreement with the experiment. For example, in the Sm(C_5_H_4_SiMe_3_)_3_^*x*–^ case, where the 4f- and 5d-orbital occupancies are well established, energy differences between the Sm^III^ (4f^5^ 5d^0^) and Sm^II^ (4f^6^ 5d^0^) L_3_-edge positions are calculated to be 6.5 eV, which is in good agreement with the measured value of 7.6 eV. Results from the B3LYP calculations modestly agree with the experimental data, while larger deviations are observed using BLYP and PBE. The two GGA functionals, BLYP and PBE, without any HF exchange give the same L_3_-edge energy difference. This comparison (BHandHLYP, B3LYP, BLYP, and PBE) unambiguously shows the importance of Hartree–Fock (HF) exchange in computationally evaluating L_3_-edge XANES spectra. This result highlights the importance of high HF exchange in correctly capturing electron transition energies and is consistent with conclusions from previous theoretical studies.^22^

Calculations on Ho(C_5_H_4_SiMe_3_)_3_^*x*–^ are similar to those from Sm(C_5_H_4_SiMe_3_)_3_^*x*–^ in that the BHandHLYP provides the best agreement with the experimental data (Table 5), *e.g.* energy differences between the Ho^III^ (4f^10^ 5d^0^) and Ho^II^ (4f^10^ 5d^1^) L_3_-edge peak maxima are calculated to be 0.7 eV and measured to be 0.5 eV. The Ho(C_5_H_4_SiMe_3_)_3_^*x*–^ calculations differ in that they invoke the Ho^II^ low energy 4f^10^ 5d^1^ ground-state electronic configuration. We note that calculations involving the higher energy 4f^11^ 5d^0^ Ho^II^ electronic configuration grossly overestimate the Ho^III^/Ho^II^ L_3_-edge energy by 6.5 eV.

**Fig. 11:**
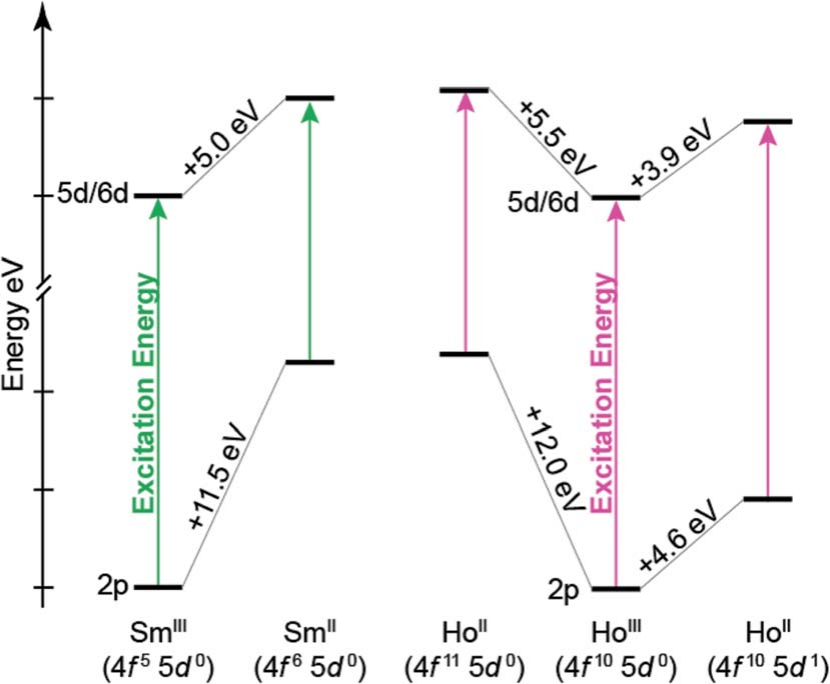
Quantitative comparison of ground-state 2p- and average 5d/6d-orbital energies from Ln(C_5_H_4_SiMe_3_)_3_^*x*–^ (Ln = Sm, Ho; *x* = 0, 1) for a variety of electronic configurations. The solid arrow represents the excitation energy associated with the Ln L_3_-edge excitation. To plot both Sm and Ho on the energy scale, the energies associated with the Ln^III^ 2p-orbitals were set to zero.

To better understand the how 4f- *versus* 5d-orbital occupancy influence Ln L_3_-XANES spectra, the ground-state 2p-orbital energies are plotted alongside the average 5d- and 6d-orbital energies in Fig. 11 for Ln(C_5_H_4_SiMe_3_)_3_^*x*–^ (Ln = Sm, Ho; *x* = 0, 1). We remind the reader that the major contributors to the Ln(C_5_H_4_SiMe_3_)_3_^*x*–^ L_3_-edge XANES spectra result from dipole allowed transitions between core 2p- and unoccupied d-orbitals. Upon reduction of Ln^III^ to Ln^II^, the 2p-, 5d-, and 6d-orbital energies increase. For both Sm and Ho, adding the electron into the 4f-shell, Ln^III^ (4f^*n*^ 5d^0^) + 1e^1–^ → Ln^II^ (4f^*n*+1^ 5d^0^), raises the Ln 2p- and 5d-/6d-orbital energies by 11.5–12.0 eV and 5.0–5.5 eV, respectively. These changes in orbital energies account for Sm^II^(C_5_H_4_SiMe_3_)_3_^1–^ L_3_-edge excitation energy being ∼7 eV less than that of Sm^III^(C_5_H_4_SiMe_3_)_3_. Adding the electron into 5d-shell, Ln^III^ (4f^*n*^ 5d^0^) + 1e^1–^ → Ln^II^ (4f^*n*^ 5d^1^), also increases the Ln 2p- and 5d-/6d-orbital energies; however, to a lesser extent. Most notably for the 2p-orbitals. For example, the Ho 2p- and 5d/6d-average orbital energies increase by 4.6 eV and 3.9 eV, respectively. This modest energy shift decreases the L_3_-edge excitation energy for Ho^II^(C_5_H_4_SiMe_3_)_3_^1–^ by <1 eV in comparison to Ho^III^(C_5_H_4_SiMe_3_)_3_. Overall, these results demonstrate that Ln 2p-electrons experienced stronger Coulomb repulsion from Ln 4f-electrons than higher lying 5d-electrons. We additionally correlate the magnitude of this repulsion with the radial distribution of the 4f- *versus* 5d-orbitals. Because the 4f-orbitals are closer to the nucleus,^31^ increased 4f-orbital occupancy destabilizes the core 2p-orbital energies to a large extent. Meanwhile, occupancy of the more diffuse 5d-orbitals has less impact on the 2p-orbital energies.

## Discussion

Herein we describe the use of XANES spectroscopy to characterize the electronic configurations of formally +2 lanthanide compounds of the general formula Ln^II^(C_5_H_4_SiMe_3_)_3_^1–^. Through comparisons with a carefully selected series of standards, including Ln^III^(C_5_H_4_SiMe_3_)_3_, our XANES results from Sm^II^(C_5_H_4_SiMe_3_)_3_^1–^, Tm^II^(C_5_H_4_SiMe_3_)_3_^1–^ and Yb^II^(C_5_H_4_SiMe_3_)_3_^1–^demonstrate that these compounds contained Ln^II^ ions with 4f^6^ 5d^0^ (Sm^II^), 4f^13^ 5d^0^ (Tm^II^), and 4f^14^ 5d^0^ (Yb^II^) electronic configurations. These results are in agreement with previously acquired spectroscopic data, *i.e.* UV-vis, magnetic susceptibility, and the Ln–C_centroid_ distances (which were ∼0.1 Å longer than the Ln^III^ analogue). Consistent with previous studies,^8a,8d^ the measurements highlight the utility of Ln L_3,2_-edge XANES spectroscopy in characterizing f-orbital occupancies from Ln^III^ (4f^*n*^ 5d^0^) and Ln^II^ (4f^*n*+1^ 5d^0^) ions. For example, changes in 4f-electron occupancy shift the Ln peak maxima in the L_3,2_-edges by approximately 7 eV. The magnitude of these shifts is impressive in comparison to transition metal K- and L-edge XANES experiments,^17,18^ where changes in d-orbital occupancies are known to shift absorption edges by only a few eV.

The Ln L-edge XANES studies from Ln(C_5_H_4_SiMe_3_)_3_^*x*–^ (Ln = Pr, Nd, Gd, Tb, Dy, Ho, and Er; *x* = 0, 1) show much smaller shifts in rising-edge energies than the samarium, thulium, and ytterbium analogues. For example, the peak maxima differences between Ln^III^(C_5_H_4_SiMe_3_)_3_ and Ln^II^(C_5_H_4_SiMe_3_)_3_^1–^ range from only 0.2 to 1.0 eV (Table 1). These values are substantially less than the 7–8 eV change expected for an increase in 4f-orbital occupancy, *i.e.* Ln^III^ (4f^*n*^ 5d^0^) + e^1–^ → Ln^II^ (4f^*n*+1^ 5d^0^). Instead, the 0.2 to 1.0 eV shifts are reminiscent of the those accompanying the reduction of Y^III^(C_5_H_4_SiMe_3_)_3_ (4d^0^) to Y^II^(C_5_H_4_SiMe_3_)_3_^1–^ (4d^1^) and Lu^III^(C_5_H_4_SiMe_3_)_3_ (4f^14^ 5d^0^) to Lu^II^(C_5_H_4_SiMe_3_)_3_^1–^ (4f^14^ 5d^1^). In these yttrium and lutetium scenarios, the increase in d-orbital occupancy shifts the peak maximum by only ~1 eV (inflection point change of 1.4 eV) and 1.9 eV, respectively. These shifts provide strong evidence that the yttrium ion in Y^II^(C_5_H_4_SiMe_3_)_3_^1–^ is best described as +2 with a 4d^1^ electronic configuration and that the lutetium ion in Lu^II^(C_5_H_4_SiMe_3_)_3_^1–^ is +2 with a 4f^14^ 5d^1^. Given that shifts from Ln(C_5_H_4_SiMe_3_)_3_^*x*–^ (Ln = Pr, Nd, Gd, Tb, Dy, Ho, and Er; *x* = 0, 1) were also small, we initially questioned the possibility that reduction of Ln^III^(C_5_H_4_SiMe_3_)_3_ (4f^*n*^ 5d^0^) generated a lanthanide ion with a 4f^*n*^ 5d^1^ electronic configuration, instead of the more typical 4f^*n*+1^ 5d^0^ configuration.

To better understand the Ln L_3_-edge XANES spectra from Ln(C_5_H_4_SiMe_3_)_3_^*x*–^ (*x* = 0, 1), DFT calculations were conducted on the Sm(C_5_H_4_SiMe_3_)_3_^*x*–^ and Ho(C_5_H_4_SiMe_3_)_3_^*x*–^ analytes. Consistent with previous reports, the ground-state DFT calculations show the electronic configurations for Sm^III^(C_5_H_4_SiMe_3_)_3_, Sm^II^(C_5_H_4_SiMe_3_)_3_^1–^, and Ho^III^(C_5_H_4_SiMe_3_)_3_ are Sm^III^ 4f^5^ 5d^0^, Sm^II^ 4f^6^ 5d^0^, and Ho^III^ 4f^10^ 5d^0^, respectively. In contrast for Ho^II^(C_5_H_4_SiMe_3_)_3_^1–^, the calculations indicate that the ground-state electronic configuration is 4f^10^ 5d^1^, with the non-bonding 5d_z2_-orbital of *a*′-symmetry being singly occupied. CASPT2/CASSCF calculations on the simplified models, Ln(C_5_H_5_)_3_^*x*–^ (Ln = Sm, Ho; *x* = 0, 1), were completely consistent with the assignments of the DFT calculations. As such the Ln L_3_-edge XANES spectra were simulated using transition dipole moment calculations for a variety of electronic configurations, spanning Ln^III^ 4f^*n*^ 5d^0^, Ln^II^ 4f^*n*+1^ 5d^0^, and Ln^II^ 4f^*n*^ 5d^1^. For both Sm and Ho, the calculations suggest that reducing Ln^III^ (4f^*n*^ 5d^0^) by adding an electron in the 4f-manifold to generate Ln^II^ (4f^*n*+1^ 5d^0^) appreciably shifts the Ln L_3_-edge by approximately 7 eV. In contrast, reducing Ln^III^ (4f^*n*^ 5d^0^) by adding an electron into the 5d-manifold to generate Ln^II^ (4f^*n*^ 5d^1^) slightly shifts the Ln L_3_-edge to lower energy (on the order of ∼1 eV).

## Concluding remarks

Our results indicate that the differences in Ln(C_5_H_4_SiMe_3_)_3_^*x*–^ (Ln = Sm, Ho; *x* = 0, 1) excitation energies stem from electron repulsion between 2p- and either 5d- or 4f-electrons (Fig. 11). For example, increases in Ln 4f-orbital occupation significantly destabilize the core 2p-orbital energy levels, which decrease the Ln L_3_-edge excitation energy by ∼7–8 eV. In contrast, increased occupancy for the more diffuse 5d-orbitals has marginal impact on core 2p-energy levels and the Ln L_3_-edge excitation energy (0.2–1.9 eV). One might describe the 4f^10^ 5d^1^ electron configuration in Ho^II^(C_5_H_4_SiMe_3_)_3_^1–^ as mimicking the 4f^10^ electronic configuration in Ho^III^(C_5_H_4_SiMe_3_)_3_, with the extra electron ‘hidden’ in a highly shielded 5d-orbital. We anticipate that this interpretation is quite general and will be used to explain the similar Ln^II^/Ln^III^ peak maxima shifts and Ln^II^/Ln^III^–C_centroid_ bond distances in the other Ln(C_5_H_4_SiMe_3_)_3_^*x*–^ (Ln = Pr, Nd, Gd, Tb, Dy, Er, and Lu; *x* = 0, 1) compounds. Hence, our current computational and spectroscopic efforts are focused on evaluating recently reported compounds that contain formally lanthanide(ii) and actinide(ii) ions.

Among the numerous examples where ligand environments with *C*_3_-symmetry have been exploited to advance transition metal and f-element chemistry,^32^ our results highlight another extraordinary property associated with a *C*_3_-ligand framework. For example, we identified that the tris-cyclopentadienyl coordination environment provides a mechanism for stabilizing Ln^II^ 4f^*n*^ 5d^1^ electronic configurations through the accessibility of a low-lying 5d-orbital of *a*′ symmetry. The results additionally suggest an electronic structure break between Tm^II^(C_5_H_4_SiMe_3_)_3_^1–^ and Dy^II^(C_5_H_4_SiMe_3_)_3_^1–^. It appears that 4f^*n*+1^ 5d^0^ electronic configurations are most stable when the reduction potentials for the lanthanide ions in Ln^II^(C_5_H_4_SiMe_3_)_3_^1–^ are less than or equal to that of Tm^II^(C_5_H_4_SiMe_3_)_3_^1–^. Meanwhile, those with reduction potentials greater than or equal to Dy^II^(C_5_H_4_SiMe_3_)_3_^1–^ are best described as 4f^*n*^ 5d^1^. While the generality of this interpretation has yet to be determined, we anticipate – based on previous studies on LnX_2_ (X = halide) – that the electronic structure breaking point is quite dynamic and can shift to higher reduction potentials, *i.e.* those of Dy^II^ and Nd^II^, depending in the ligand environment. Our current efforts are focused on identifying the implications of these results on lanthanide reactivity.

## Experimental

### Sample preparation

The analytes were synthesized at the University of California in Irvine CA with rigorous exclusion of air and moisture.^1c,8a^ The Ln^III^(C_5_H_4_SiMe_3_)_3_,^33^ Ln^II^(C_5_H_4_SiMe_3_)_3_^1–^,^1,8a^ Sm^II^(C_5_Me_5_)_2_(THF)_2_,^34^ Sm^III^[N(SiMe_3_)_2_]_3_,^35^ Sm^II^[N(SiMe_3_)_2_]_2_(THF)_2_,^36^ Tm^II^I_2_(THF)_2_,^37^ and Tm^III^I_3_(THF)_3.5_ (ref. 38) were prepared as previously described. Analytes were sealed in ampoules and transported in a cooler filled with dry ice to the Stanford Synchrotron Radiation Lightsource (SSRL) where they were stored at –80 °C. Three hours prior to analysis by XAFS, the lanthanide samples were transferred into an argon filled glovebox. The samples were kept cold by preparing them on an aluminum block, which had been plumbed to accommodate flowing helium gas cooled from a dry ice/ethanol bath. Note, all equipment (including the holder, spatulas, wrenches, boron nitride, *etc.*) were cooled on the block prior to sample preparation. Samples were diluted with boron nitride, which had been dried at elevated temperature (200 °C) under vacuum (10^–3^ Torr) for 48 hours. A mixture of the analyte and BN were weighed out, such that the edge jump for the absorbing atom was calculated to be at ∼1 absorption length in transmission (between 8 to 30 mg of sample and ∼50 mg of BN). Samples were ground using a Wig-L-Bug®, a Teflon bead, and a polycarbonate capsule. The finely ground powders were pressed as a pellet into a slotted aluminum sample holder. These precautions were taken to minimize self-absorption. The holder was equipped with Kapton windows (1 mil), one was fixed with super glue and the other was Kapton tape. For Pr, Nd, Sm, Gd, Tb, Dy, Y, Ho, Er, Tm, Yb and Lu analytes, the holder was brought out of the glovebox, immediately submerged in liquid nitrogen for transportation to the beam line, and loaded into the cryostat. The cryostat was immediately evacuated and attached to the beamline 11-2 XAFS rail and cooled with either liquid nitrogen or liquid helium.

### Data acquisition

The cryostat was attached to the beamline 11-2 XAFS rail (SSRL), which was equipped with three ionization chambers through which nitrogen gas was continually flowed. One chamber (10 cm) was positioned before the cryostat to monitor the incident radiation (*I*_0_). The second chamber (30 cm) was positioned after the cryostat so that sample transmission (*I*_1_) could be evaluated against *I*_0_ and so that the absorption coefficient (*μ*) could be calculated as ln(*I*_0_/*I*_1_). The third chamber (*I*_2_; 30 cm) was positioned downstream from *I*_1_ so that the XANES of a calibration foil could be measured against *I*_1_. A potential of 1600 V were applied in series to the ionization chambers.

Samples were calibrated to the energy of the first inflection point of a calibration foil, whose spectrum was measured *in situ* from the sample using the transmitted portion of the beam. The measurements were calibrated as follows. The Y K-edges were calibrated to the Y K-edge (17 038.4 eV) of an yttrium foil. The Lu L_3_-edge to the Cu K-edge of a copper foil at 8979 eV. The Er and Yb L_3_-edges to the Ni K-edge of a nickel foil at 8333 eV. The Tm L_3_-edges were calibrated to the Ho L_3_-edge at 8070.1 eV. The Dy L_3_-edge was calibrated to the Dy L_3_-edge of a dysprosium foil at 7790.0 eV. The Ho L_3_-edges to the Co K-edge of a cobalt foil at 7709 eV. Sm, Gd, and Tb L-edges to the Fe K-edge of an iron foil at 7111 eV. The Pr, and Nd L-edges to the Cr K-edge of a chromium foil at 5989 eV.

The X-ray absorption near edge spectra (XANES) were measured at the SSRL, under dedicated operating conditions (3.0 GeV, 5%, 500 mA using continuous top-off injections) on end station 11-2. This beamline, which was equipped with a 26-pole, 2.0 tesla wiggler, utilized a liquid nitrogen-cooled double-crystal Si[220] monochromator and employed collimating and focusing mirrors. A single energy was selected from the white beam with a liquid-N_2_-cooled double-crystal monochromator utilizing Si[220] (*φ* = 0) crystals. Harmonic rejection was achieved by detuning the second crystal of the monochromator by 50% at ∼600 eV above the absorbing edge. The vertical slit sizes were 1 mm and the beam was unfocused.

### Data analysis

Data manipulations and analyses were conducted as previously described.^39^ Energy calibrations were conducted externally using the first inflection point of the rising edge of the calibration spectrum. Data were analyzed by fitting a line to the pre-edge region, which was subsequently subtracted from the experimental data to eliminate the background of the spectrum. The data were normalized by fitting a first-order polynomial to the post-edge region of the spectrum and setting the edge jump at to an intensity of 1.0.

### UV-visible spectroscopy

Prior to transporting the Ho^II^(C_5_H_4_SiMe_3_)_3_^1–^ samples to the synchrotron, the compound was characterized by UV-vis, as previously reported.^8^ The sample was first prepared for XANES analysis in an argon-filled glovebox by finely grinding Ho^II^(C_5_H_4_SiMe_3_)_3_^1–^ (19.4 mg) with cold anhydrous boron nitride, BN (60.6 mg) for 2 min in polystyrene canisters with plexiglass pestles using a Wig-L-Bug® grinder to obtain a homogeneous fine powder. The sample was loaded within a slotted aluminum holder, whose slot dimensions were 5 × 20 × 1 mm. The holder was equipped with Kapton tape windows (1 mL). This holder was nested within an additional holder, also equipped with Kapton windows (1 mL) that were sealed with indium wire gaskets. This holder is well established as providing robust exclusion of air and moisture. The sample holder was placed on the rail at SSRL's beam line 11-2 and the Ho L_3_-edge spectrum obtained in transition mode at room temperature. After data collection the holder was returned to the glovebox and disassembled. The Ho^II^(C_5_H_4_SiMe_3_)_3_^1–^ and BN mixture was transferred to a Teflon sealable quartz cuvette with THF (dried over Na/K alloy and benzophenone). The sample was again removed from the glovebox and analyzed using a CARY 50 spectrometer. The UV-vis data were background-subtracted. Owing to the suspended BN, a constant 1.15 absorption value was subsequently subtracted to set the background to zero.

### Density functional calculations

Ground-state electronic structure calculations were performed on the Ln(C_5_H_4_SiMe_3_)_3_^*x*–^ (Ln = Sm, Ho; *x* = 0, 1) using the generalized gradient approximation (GGA) with the PBE exchange–correlation functional^40^ as implemented in the Amsterdam Density Functional (ADF 2014.11).^41–43^ For geometry optimization, the Slater basis sets with the quality of triple-ζ plus one polarization functions (TZP)^44^ were used, with the frozen core approximation applied to the inner shells [1s^2^–4d^10^] for Sm and Ho, [1s^2^] for C, [1s^2^–2p^6^] for Si. All electron TZ2P basis sets were used for spectroscopic simulation by employing the PBE,^40^ BLYP,^45,46^ B3LYP,^45,46^ and BHandHLYP^47,48^ functionals. The latter three functionals combine the LYP^46^ GGA for correlation with three different approximations for exchange, *i.e.*, Becke's GGA (B)^45^ for exchange, the Becke's three-parameter (B3)^47^ hybrid functional including 20% HF exact exchange, and the half-and-half hybrid containing 50% HF exact exchange.^48^ The B3LYP and BHandHLYP functionals were chosen because they give good performance in excitation energy of charge-transfer states and were commonly used.^22a,49,50^ The BLYP was employed together with B3LYP and BHandHLYP to study the impact of the percentage of HF exchange on the excitation energy and spectral shape. The scalar relativistic (SR) effects were taken into account by the zero-order regular approximation (ZORA).^51^ Geometries were fully optimized without symmetry at the SR-ZORA level with the gradient convergence of 10^–5^, and frequency calculations were carried out to verify the local minimum on the potential energy surface. In the ground-state electronic structure calculations for Ln(C_5_H_4_SiMe_3_)_3_^*x*–^ (Ln = Sm, Ho; *x* = 0, 1), the high-spin multiplicity was used for each electron configuration. Specifically, Sm^III^ (4f^5^ 5d^0^) had a ground sextet state, and Sm^II^ (4f^6^ 5d^0^) had a ground septet state; Ho^III^ (4f^10^ 5d^0^) has ground quintet state, and Ho^II^ (4f^10^ 5d^1^) had a ground sextet state, and Ho^II^ (4f^11^ 5d^0^) had ground quartet state (Table 3).

### DFT-simulation of Ln L_3_-edge XANES spectra

The L_3_-edge XANES spectra from Ln(C_5_H_4_SiMe_3_)_3_^*x*–^ (Ln = Sm, Ho; *x* = 0, 1) were simulated as the Kohn–Sham orbital energy differences, *i.e.*, the energy difference between an occupied orbital and a virtual orbital of the ground-state. For a specific core excitation, the oscillator strength was calculated from the transition dipole approximation between this occupied orbital and the virtual orbital. The core electron excitation was calculated originating from Ln 2p dominated MOs to virtual MOs at the DFT/PBE optimized ground-state geometry. All other excitations from orbitals between the Ln 2p and HOMOs were excluded by restricting the energy range of the occupied orbitals involved in the excitations, so that only excitations from Ln 2p core levels to virtual MOs were allowed. The relaxation due to the core hole was assumed constant. All the calculated transition intensities were evenly broadened with a Gaussian function of full-width at half-maximum of 1.7 eV (*i.e.*, peak width) to emulate the experimental spectra.

### FEFF spectral simulations

The Ln(C_5_H_4_SiMe_3_)_3_^*x*–^ (Ln = Sm and Tm; *x* = 0, 1) Sm and Tm L_3_-edge and Y(C_5_H_4_SiMe_3_)_3_^*x*–^ (*x* = 0, 1) Y K-edge XANES spectra and the angular momentum projected density of states were calculated with the FEFF9.6 *ab initio* quantum chemical code based on the multiple scattering theory (see ESI†).^14^ The potentials of free atoms were calculated with a relativistic Dirac–Fock atom code part of FEFF9.6. The scattering potentials were calculated self-consistently by overlapping the free atomic densities in the muffin tin approximation within a cluster of 334 atoms (SCF card; UNFREEZF card was not included). The energy dependent exchange Hedin–Lundquist potential was used for the fine structure and the atomic background (EXCHANGE card). The full multiple scattering XANES spectra were calculated for an atomic cluster of 334 atoms centered on the absorbing Sm/Tm/Y atom (FMS and XANES cards). Best agreement between calculation and experiment was found by applying “COREHOLE FSR” option to screen the 2p_3/2_ (Sm/Tm) or 1s (Y) core-holes. The FOLP card (FOLP 1 1.07) was used for calculating the Sm spectra, as the overlap of the muffin tin radii was reported to be too large by the program. This value was chosen as it was found for the calculations of the Tm and Y spectra. We have obtained comparable results (not shown here) for Tm by including the f valence states in the self-consistent calculations of the scattering potentials (UNFREEZF card).

### CASPT2/CASSCF calculations

Using the complete-active-space multi-configuration approach with second-order perturbation theoretical correction (CASPT2)^52,53^ implemented in Molpro 2015.1 program, *ab initio* WFT calculations were performed.^54,55^ To reduce the computational cost, CASPT2/CASSCF calculations were carried out on the ground-states and low excited-states of the simplified Ln(C_5_H_5_)_3_^*x*–^ (Ln = Sm, Ho; *x* = 0, 1) complexes. The DFT/PBE optimized geometries of Ln(C_5_H_4_SiMe_3_)_3_^*x*–^ were used in the calculations. Here the original SiMe_3_ substituents, ancillary groups, were replaced with protons having C–H bond lengths of 1.088 Å. For Ho(C_5_H_5_)_3_^1–^, two geometries derived from Ho^II^ (4f^11^ 5d^0^) and Ho^II^ (4f^10^ 5d^1^) were used. We applied the cc-pVDZ basis sets for H and C,^56^ Stuttgart energy-consistent relativistic pseudopotentials ECP28MWB,^57,58^ and the corresponding ECP28MWB-SEG basis for Sm and Ho. Although attempts to include all the seven 4f- and five 5d-orbitals into active space were made, the converged CASSCF results showed that for Sm(C_5_H_5_)_3_^*x*–^ (*x* = 0, 1) the five 5d-orbitals are not correlated and were removed out of active space. In contrast for Ho(C_5_H_5_)_3_^*x*–^ (*x* = 0, 1), only the 5d_z2_-orbital remained in the active space. Therefore, the active space was adjusted to include all the 4f-orbitals for Sm(C_5_H_5_)_3_^*x*–^ (*x* = 0, 1) and additionally the 5d_z2_ – character orbital for Ho(C_5_H_5_)_3_^*x*–^ (*x* = 0, 1). In the CASPT2 calculations, the ionization-potential/electron-affinity corrected zeroth-order Hamiltonian was used with an IPEA shift of 0.25 a.u.^59^ The 1s-core orbitals of the C atoms, and 4s-, 4p-, 4d-orbitals of the Sm and Ho atoms were kept frozen in the CASPT2 calculations.
