# Supplementary material for: Evaluating the electronic structure of formal LnII ions in LnII(C5H4SiMe3)3 1– using XANES spectroscopy and DFT calculations
Source: Chem Sci. 2017 Jun 30;8(9):6076–91. doi: 10.1039/c7sc00825b (PMC5625586; doi:10.1039/c7sc00825b)
Supplement: Supplementary file 1 [file SC-009-C7SC00825B-s001.pdf]

Electronic Supplementary Material (ESI) for Chemical Science.
This journal is © The Royal Society of Chemistry 2017

Supplemental Information

Evaluating the Electronic Structure of Formal Ln^{II} Ions in $Ln^{II}(C_5H_4SiMe_3)_3^{1-}$ Using XANES Spectroscopy and DFT Calculations

Megan E. Fieser,[1,a] Maryline G. Ferrier,[2,a] Jing Su,[2,a] Enrique Batista,[2]* Samantha K. Cary,[2] Jonathan W. Engle,[2,3] William J. Evans,[1]* Juan S. Lezama Pacheco,[4] Stosh A. Kozimor,[2]* Angela C. Olson,[2] Austin J. Ryan,[1] Benjamin W. Stein,[2] Gregory L. Wagner,[2] David H. Woen,[1] Tonya Vitova,[5] and Ping Yang[2]*

[1] *University of California, Irvine CA 92697*

[2] *Los Alamos National Laboratory, Los Alamos, NM 87545*

[3] *University of Wisconsin, Madison, Wisconsin 53711*

[4] *Stanford University, Palo Alto, CA 94305*

[5] *Karlsruhe Institute of Technology, Institute for Nuclear Waste Disposal, P.O. Box 3640, 76021 Karlsruhe, Germany*

[a] M. E. Fieser, M. G. Ferrier, J. Su contributed equally to this work.

* To whom correspondence should be addressed, stosh@lanl.gov; wevans@uci.edu; erb@lanl.gov; pyang@lanl.gov

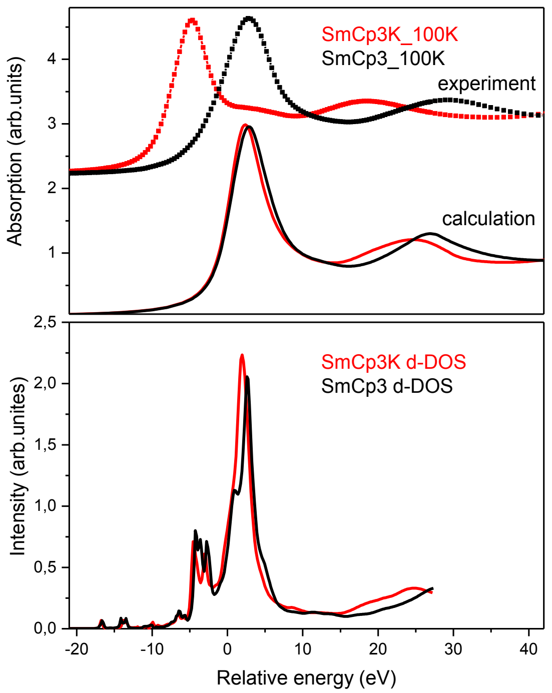

Figure S1: *Top*-The experimental and calculated (FEFF9.6 code) Sm L_3-edge XANES spectra from $Sm(C_3H_4SiMe_3)_3^{x-}$ (x = 0, 1). *Bottom*-The calculated d-DOS (bottom) from $Sm(C_3H_4SiMe_3)_3^{x-}$ (x = 0, black trace; x = 1 red trace).

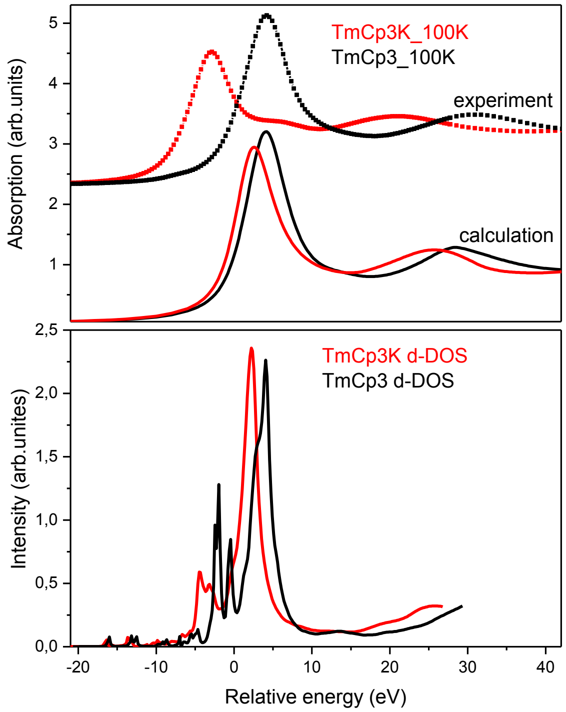

Figure S2: *Top*-The experimental and calculated (FEFF9.6 code) Tm L_3-edge XANES spectra from $Tm(C_3H_4SiMe_3)_3^{x-}$ (x = 0, 1). *Bottom*-The calculated d-DOS (bottom) from $Tm(C_3H_4SiMe_3)_3^{x-}$ (x = 0, black trace; x = 1 red trace).

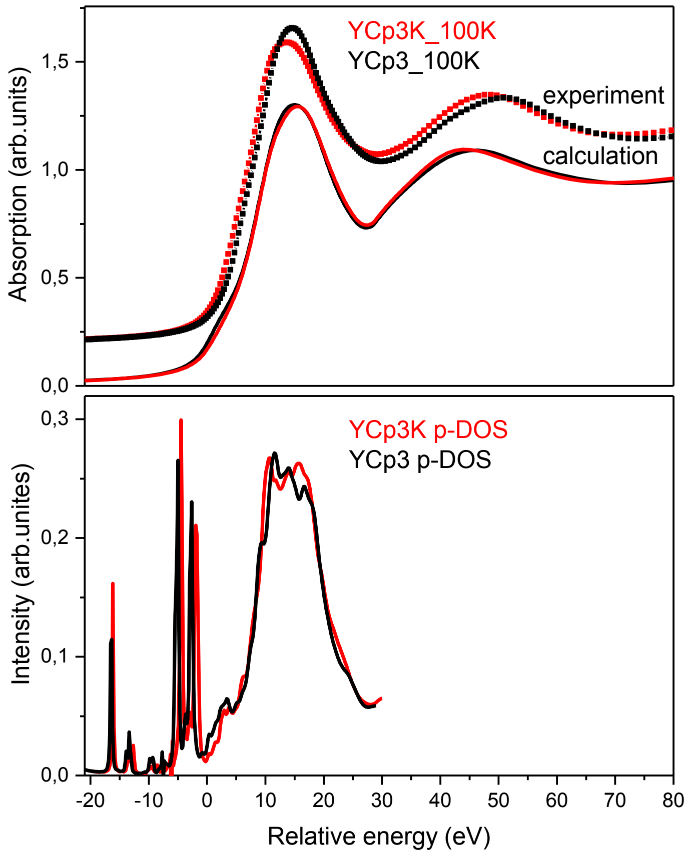

Figure S3: *Top*-The experimental and calculated (FEFF9.6 code) Y K-edge XANES spectra from $Y(C_3H_4SiMe_3)_3^{x-}$ (x = 0, 1). *Bottom*-The calculated d-DOS (bottom) from $Y(C_3H_4SiMe_3)_3^{x-}$ (x = 0, black trace; x = 1 red trace).

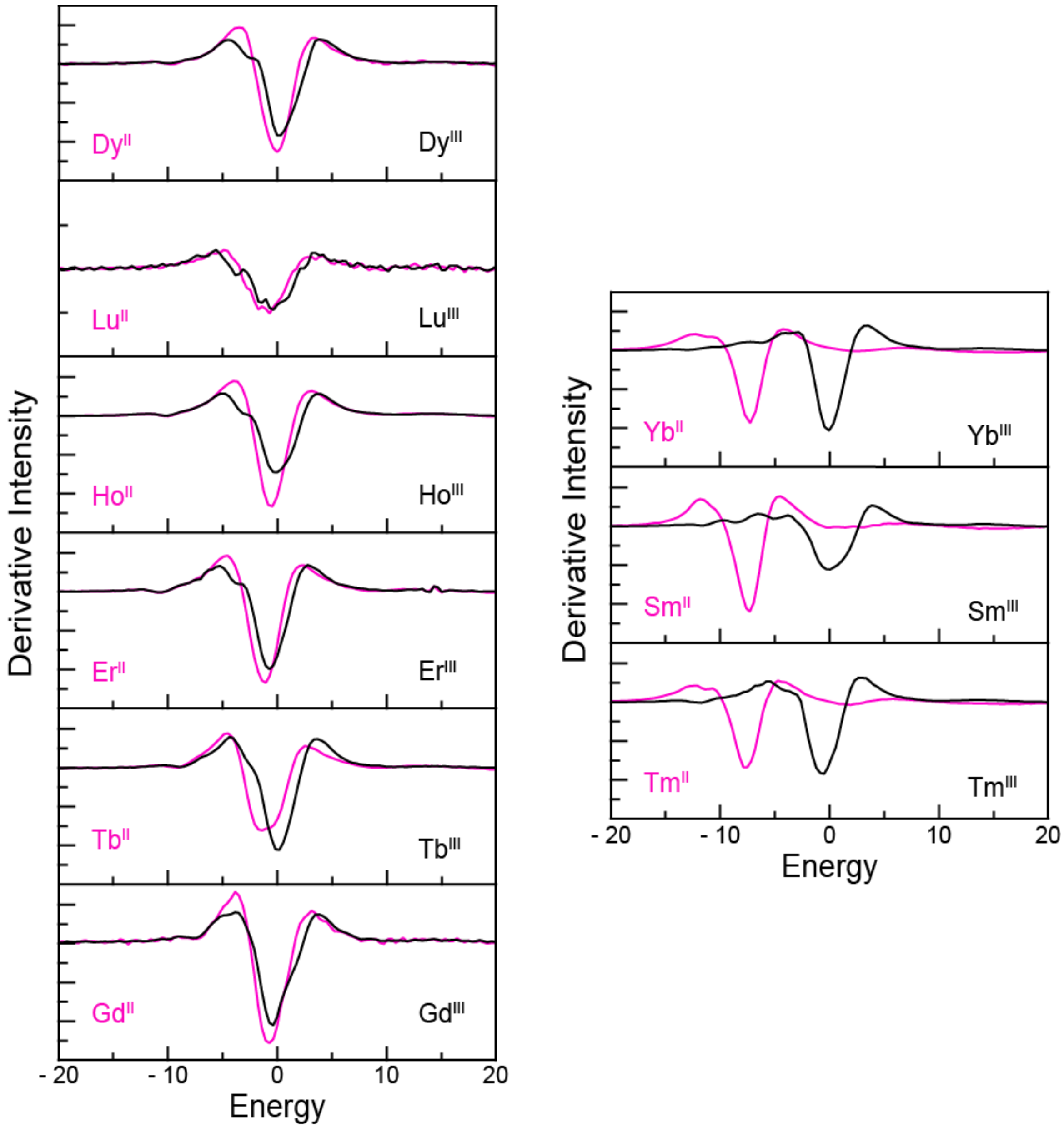

Figure S4. Second derivatives of the L_3-edge XANES measurements obtained from $Ln^{III}(C_5H_4SiMe_3)_3$ (black traces) and $[K(2.2.2\text{-cryptand})][Ln^{II}(C_5H_4SiMe_3)_3]$ (pink trace) for Ln = Dy, Lu, Ho, Er, Tb, Gd on the left and Yb, Sm, Tm on the right. Note, even though the $Lu(C_5H_4SiMe_3)_3^{x-}$ (x = 0, 1) data is quite noisy, the second derivatives indicate that a pre-edge feature is well resolved from the rising edge.

Branching ratios were determined as described previously using a graphical approach based on the integration of the second-derivative of the data as defined by $A_5/(A_5+A_4)$. Here, A_5 and A_4 are the total areas under the second derivative of the L_3 and L_2-edge peaks, respectively. The branching ratio performed on all lanthanide compounds gave similar results 0.62 – 0.66, see Table S1.

Table S1. Branching ratio for $Ln^{III}(C_5H_4SiMe_3)$, [K(2.2.2-cryptand)][$Ln^{II}(C_5H_4SiMe_3)$] for Ln = Sm, Tb, Ho, Tm, $Sm^{II}(C_5Me_5)_2(THF)_2$, $TmI_2(THF)_3$, and $TmI_3(THF)_{3.5}$.

Compound	Branching Ratio	STDEV	STDEV (%)
$Sm^{II}(C_5H_4SiMe_3)_3{}^{1-}$	0.655	0.00073	0.11
$Sm^{III}(C_5H_4SiMe_3)_3$	0.626	0.00017	0.03
$Sm^{II}(C_5Me_5)_2THF_2$	0.647	0.00076	0.12
$Tb^{II}(C_5H_4SiMe_3)_3{}^{1-}$	0.639	0.0008	0.13
$Tb^{III}(C_5H_4SiMe_3)_3$	0.656	0.0004	0.06
$Ho^{II}(C_5H_4SiMe_3)_3{}^{1-}$	0.611	0.0044	0.72
$Ho^{III}(C_5H_4SiMe_3)_3$	0.639	0.003	0.05
$Tm^{II}(C_5H_4SiMe_3)_3{}^{1-}$	0.647	0.00847	1.31
$Tm^{III}(C_5H_4SiMe_3)_3$	0.641	0.00112	0.17
$Tm^{II}I_2(THF)_3$	0.620	0.0024	0.39
$Tm^{III}I_3(THF)_{3.5}$	0.642	0.00147	0.23

Table S2. The DFT-PBE//TZP optimized ground-state geometrical coordinates (in Å) and DFT-XC//TZ2P (XC=PBE, BLYP, B3LYP, BHandHLYP) single-point calculated total bonding energies (*E*, in kcal/mol) of $Ln(C_5H_4SiMe_3)_3^{x-}$ (Ln = Sm, Ho; x = 0, 1).

$Sm(C_5H_4SiMe_3)_3$ ($4f^55d^0$, sextet state) *E(PBE)*=-8935.82; *E(BLYP)*= -8485.47 *E(B3LYP)*= -10154.16; *E(BHandHLYP)*= -12489.41	$Sm(C_5H_4SiMe_3)_3^-$ ($4f^65d^0$, septet state) *E(PBE)*= -8971.53; *E(BLYP)*= -8520.87 *E(B3LYP)*= -10191.16; *E(BHandHLYP)*= -12515.81
1.Sm 0.588630 6.013376 3.465276	1.Sm 0.491007 5.987784 3.468516
2.Si 2.095627 8.909309 0.888513	2.Si 2.288933 8.802001 0.896949
3.Si -2.198432 9.156150 4.382272	3.Si -2.260108 9.204081 4.383259
4.Si 2.009055 2.651269 5.329473	4.Si 2.272345 2.728553 5.296229
5.C 0.835056 6.222294 0.682105	5.C 0.808983 6.266185 0.632215
6.H -0.038290 6.556995 0.126100	6.H -0.043787 6.698079 0.110766
7.C 1.141731 4.873379 0.991244	7.C 0.999507 4.889700 0.890383
8.H 0.552803 4.001555 0.708795	8.H 0.332162 4.082491 0.588258
9.C 2.376805 4.859275 1.690104	9.C 2.234146 4.743005 1.586753
10.H 2.905759 3.974881 2.041347	10.H 2.682177 3.801881 1.903158
11.C 2.818989 6.200032 1.814616	11.C 2.792366 6.030521 1.755992
12.H 3.750888 6.513957 2.281957	12.H 3.742824 6.247410 2.242034
13.C 1.864635 7.068794 1.197908	13.C 1.911830 7.003583 1.173603
14.C 3.727013 9.170631 -0.033349	14.C 3.860034 8.985939 -0.165734
15.H 3.723587 8.632314 -0.992553	15.H 3.728549 8.483935 -1.136400
16.H 4.578502 8.797330 0.555166	16.H 4.722507 8.518193 0.333697
17.H 3.900593 10.238214 -0.238719	17.H 4.105561 10.043935 -0.352042
18.C 0.665975 9.559967 -0.163503	18.C 0.863890 9.639681 -0.036443
19.H -0.307524 9.425158 0.329929	19.H -0.078286 9.578162 0.527064
20.H 0.629402 9.042352 -1.133733	20.H 0.706928 9.158738 -1.014050
21.H 0.792284 10.635029 -0.362639	21.H 1.083577 10.704271 -0.213064
22.C 2.190192 9.862932 2.527612	22.C 2.620106 9.749099 2.512678
23.H 1.265745 9.775097 3.118853	23.H 1.738299 9.738820 3.171540
24.H 2.361979 10.935160 2.345295	24.H 2.888493 10.799690 2.312809
25.H 3.023292 9.495571 3.146193	25.H 3.450202 9.286217 3.068743
26.C -1.928040 6.227477 4.696173	26.C -2.108116 6.283096 4.681453
27.H -1.958783 6.243498 5.784156	27.H -2.111811 6.285639 5.770857
28.C -1.977665 5.060884 3.894927	28.C -2.188634 5.129683 3.867846
29.H -2.050948 4.036288 4.258386	29.H -2.276767 4.101144 4.220650
30.C -1.956501 5.466686 2.535110	30.C -2.158849 5.550244 2.507337
31.H -2.006139 4.810604 1.668016	31.H -2.204731 4.901111 1.633739
32.C -1.888274 6.883425 2.508961	32.C -2.049144 6.958781 2.492797
33.H -1.891728 7.497079 1.609796	33.H -2.013050 7.580223 1.598657
34.C -1.864624 7.380175 3.850660	34.C -2.009521 7.442303 3.845884
35.C -3.972381 9.245775 5.038102	35.C -4.105699 9.514367 4.751693
36.H -4.689016 8.922077 4.268870	36.H -4.715765 9.302065 3.860417
37.H -4.231678 10.272652 5.339055	37.H -4.293314 10.556007 5.059290

38.H -4.104800 8.591699 5.912913<br>39.C -1.035326 9.741490 5.759407<br>40.H 0.020115 9.734950 5.448974<br>41.H -1.129940 9.113472 6.658147<br>42.H -1.291005 10.772109 6.051024<br>43.C -2.041910 10.317025 2.896505<br>44.H -1.021646 10.341705 2.487632<br>45.H -2.304095 11.343859 3.194073<br>46.H -2.726357 10.022317 2.086909<br>47.C 2.860338 5.478962 5.009670<br>48.H 3.781489 5.241470 4.481891<br>49.C 2.446011 6.777616 5.394485<br>50.H 2.994107 7.703363 5.223004<br>51.C 1.214800 6.651304 6.089974<br>52.H 0.649053 7.462784 6.542544<br>53.C 0.876405 5.275428 6.123186<br>54.H -0.000362 4.854871 6.612606<br>55.C 1.886116 4.525527 5.441913<br>56.C 3.778210 2.169981 4.865650<br>57.H 3.879623 1.074928 4.821191<br>58.H 4.497437 2.546864 5.607701<br>59.H 4.068195 2.569232 3.882093<br>60.C 1.555276 1.902539 7.006064<br>61.H 0.522159 2.154902 7.288926<br>62.H 2.220418 2.277507 7.797603<br>63.H 1.636236 0.804964 6.980203<br>64.C 0.817767 1.943234 4.029830<br>65.H -0.229235 2.184983 4.268612<br>66.H 0.900947 0.845694 3.992783<br>67.H 1.033944 2.323218 3.019520	38.H -4.455554 8.849895 5.556658<br>39.C -1.309440 9.645718 5.967543<br>40.H -0.222770 9.564389 5.817877<br>41.H -1.584297 8.974998 6.796230<br>42.H -1.539144 10.677081 6.280754<br>43.C -1.758829 10.426409 3.019745<br>44.H -0.683705 10.367375 2.796875<br>45.H -1.985719 11.459910 3.326787<br>46.H -2.308448 10.225909 2.087122<br>47.C 2.808956 5.629720 5.148046<br>48.H 3.757775 5.532842 4.623075<br>49.C 2.239386 6.841002 5.600259<br>50.H 2.676077 7.834450 5.495916<br>51.C 1.005860 6.531421 6.241396<br>52.H 0.329463 7.247439 6.704221<br>53.C 0.818962 5.133592 6.173939<br>54.H -0.030851 4.589898 6.587141<br>55.C 1.933511 4.545714 5.482696<br>56.C 3.988061 2.440310 4.533910<br>57.H 4.184247 1.363847 4.408612<br>58.H 4.774349 2.856267 5.182106<br>59.H 4.079096 2.918827 3.547921<br>60.C 2.244568 1.866862 6.994470<br>61.H 1.266426 1.998139 7.482292<br>62.H 3.006355 2.300648 7.659928<br>63.H 2.437732 0.785730 6.903096<br>64.C 0.968336 1.846434 4.224962<br>65.H -0.040914 1.997756 4.639101<br>66.H 1.154020 0.760933 4.178853<br>67.H 0.967757 2.235875 3.195281
$Ho(C_5H_4SiMe_3)_3$ ($4f^{10}5d^0$, quintet state)<br>*E(PBE)*=-8854.32; *E(BLYP)*=-8406.99<br>*E(B3LYP)*=-10049.08;*E(BHandHLYP)*=-12349.56	$Ho(C_5H_4SiMe_3)_3^-$ ($4f^{10}5d^1$, sextet state)<br>*E(PBE)*=-8868.29; *E(BLYP)*=-8417.36<br>*E(B3LYP)*=-10063.06; *E(BHandHLYP)*= -12359.95
1.Ho 0.587092 6.052398 3.479993<br>2.Si 2.066086 8.874173 0.892095<br>3.Si -2.163198 9.153958 4.366358<br>4.Si 2.020467 2.645229 5.278073<br>5.C 0.793492 6.185649 0.751886<br>6.H -0.078669 6.507602 0.187625<br>7.C 1.097480 4.845736 1.095859<br>8.H 0.510842 3.966948 0.832471<br>9.C 2.333880 4.847452 1.794800<br>10.H 2.860656 3.970973 2.166399<br>11.C 2.776041 6.189040 1.888816<br>12.H 3.706731 6.515331 2.349035<br>13.C 1.818513 7.045259 1.256252<br>14.C 3.696574 9.087403 -0.043546<br>15.H 3.682299 8.521933 -0.986870	1.Ho 0.621110 6.020346 3.421939<br>2.Si 2.158731 9.024863 1.014691<br>3.Si -2.240234 8.991296 4.320680<br>4.Si 1.820920 2.661707 5.468456<br>5.C 0.937327 6.352483 0.715244<br>6.H 0.059593 6.694985 0.171639<br>7.C 1.280308 4.999452 0.948372<br>8.H 0.717094 4.125742 0.624398<br>9.C 2.502152 4.980133 1.672077<br>10.H 3.027961 4.089793 2.010396<br>11.C 2.903853 6.317426 1.885652<br>12.H 3.802992 6.627402 2.416345<br>13.C 1.928852 7.204859 1.317705<br>14.C 3.703265 9.293156 -0.073428<br>15.H 3.602863 8.750502 -1.025974

16.H	4.545500	8.719995	0.552324	16.H	4.604324	8.911069	0.430867
17.H	3.883283	10.146325	-0.279973	17.H	3.865322	10.360007	-0.297400
18.C	0.639756	9.507725	-0.174025	18.C	0.676609	9.717454	0.051156
19.H	-0.333982	9.393071	0.323786	19.H	-0.266316	9.579345	0.598204
20.H	0.598416	8.965095	-1.130279	20.H	0.583677	9.208715	-0.920901
21.H	0.773737	10.576254	-0.401271	21.H	0.802682	10.794521	-0.140929
22.C	2.180631	9.872643	2.502725	22.C	2.442679	10.043019	2.592413
23.H	1.260258	9.807631	3.102897	23.H	1.583199	9.960376	3.273686
24.H	2.360202	10.938034	2.290400	24.H	2.612616	11.106759	2.355576
25.H	3.015422	9.514949	3.124820	25.H	3.326504	9.671152	3.134131
26.C	-1.861348	6.235932	4.714409	26.C	-1.880652	6.087230	4.568860
27.H	-1.888254	6.268245	5.801573	27.H	-1.922207	6.077596	5.655931
28.C	-1.894842	5.057046	3.930883	28.C	-1.878609	4.938696	3.736327
29.H	-1.958322	4.037287	4.308462	29.H	-1.915692	3.902812	4.067676
30.C	-1.887539	5.444435	2.564250	30.C	-1.865667	5.386836	2.388170
31.H	-1.934341	4.774851	1.708056	31.H	-1.844022	4.749637	1.505453
32.C	-1.833754	6.859494	2.517162	32.C	-1.839555	6.798730	2.394545
33.H	-1.845660	7.461371	1.610638	33.H	-1.818093	7.433694	1.510399
34.C	-1.805362	7.376445	3.853383	34.C	-1.828207	7.268669	3.752030
35.C	-3.936969	9.223156	5.024438	35.C	-4.094814	9.068340	4.766125
36.H	-4.650213	8.883273	4.259004	36.H	-4.711390	8.800082	3.894375
37.H	-4.210524	10.248569	5.317668	37.H	-4.393380	10.073829	5.104483
38.H	-4.058627	8.574207	5.904585	38.H	-4.330402	8.352403	5.568782
39.C	-1.006106	9.768759	5.735672	39.C	-1.295936	9.539550	5.873847
40.H	0.050422	9.760458	5.429046	40.H	-0.218838	9.622976	5.668392
41.H	-1.101150	9.157687	6.645982	41.H	-1.429169	8.821755	6.697796
42.H	-1.266654	10.803544	6.007556	42.H	-1.662466	10.520186	6.218834
43.C	-2.028107	10.303118	2.869650	43.C	-1.951970	10.282107	2.960935
44.H	-1.008515	10.345034	2.460955	44.H	-0.885763	10.374602	2.712101
45.H	-2.311322	11.327032	3.157913	45.H	-2.311289	11.268682	3.294712
46.H	-2.706230	9.987246	2.062701	46.H	-2.496510	10.021541	2.040387
47.C	2.821327	5.490930	4.977881	47.C	2.805247	5.401232	4.948568
48.H	3.751449	5.273945	4.458242	48.H	3.695131	5.099103	4.400319
49.C	2.385461	6.776825	5.377174	49.C	2.480453	6.729428	5.314182
50.H	2.924408	7.712457	5.231698	50.H	3.065556	7.620813	5.093036
51.C	1.147511	6.621266	6.059876	51.C	1.248900	6.698372	6.020623
52.H	0.567056	7.417995	6.519433	52.H	0.728345	7.559701	6.432556
53.C	0.827287	5.242527	6.062933	53.C	0.818092	5.353628	6.082430
54.H	-0.048244	4.800538	6.534164	54.H	-0.090977	5.003420	6.566637
55.C	1.850851	4.517987	5.373710	55.C	1.759924	4.517378	5.388461
56.C	3.802487	2.206371	4.818593	56.C	2.696196	1.880333	3.976026
57.H	3.933087	1.113800	4.791774	57.H	2.796846	0.791816	4.116101
58.H	4.510823	2.613573	5.555243	58.H	3.707271	2.295742	3.844086
59.H	4.084101	2.596738	3.829144	59.H	2.130242	2.062636	3.051169
60.C	1.602060	1.912687	6.971568	60.C	2.801364	2.117768	7.013998
61.H	0.563196	2.136917	7.257197	61.H	2.331053	2.512109	7.928058
62.H	2.259757	2.323142	7.751693	62.H	3.829325	2.510714	6.976408
63.H	1.718046	0.817955	6.964892	63.H	2.856132	1.020409	7.100618
64.C	0.843975	1.877084	4.002111	64.C	0.086608	1.905272	5.626786
65.H	-0.207319	2.097676	4.241767	65.H	-0.464683	2.345068	6.472028

66.H 0.957283 0.781743 3.990408<br>67.H 1.043419 2.243436 2.984082	66.H 0.152341 0.818521 5.796231<br>67.H -0.499126 2.079889 4.712851
$Ho(C_5H_4SiMe_3)_3^-$ ($4f^{11}5d^0$, quartet state)<br>*E(PBE)*=-8883.41; *E(BLYP)*=-8436.21<br>*E(B3LYP)*=-10064.88; *E(BHandHLYP)*=12332.83	
1.Ho 0.560913 6.029876 3.471738<br>2.Si 2.191469 8.840747 0.902511<br>3.Si -2.257497 9.114345 4.405883<br>4.Si 2.070488 2.718803 5.290380<br>5.C 0.823075 6.232947 0.668127<br>6.H -0.046906 6.612968 0.135765<br>7.C 1.080538 4.874384 0.953740<br>8.H 0.453408 4.029228 0.670398<br>9.C 2.317437 4.800183 1.656290<br>10.H 2.803469 3.888916 2.002153<br>11.C 2.810691 6.114720 1.808013<br>12.H 3.746738 6.387445 2.292835<br>13.C 1.882169 7.033598 1.212541<br>14.C 3.748238 9.059939 -0.172515<br>15.H 3.627284 8.539870 -1.135070<br>16.H 4.627944 8.627491 0.328478<br>17.H 3.958065 10.122526 -0.375362<br>18.C 0.731583 9.611530 -0.033350<br>19.H -0.201901 9.538148 0.542926<br>20.H 0.577781 9.100074 -0.995971<br>21.H 0.918117 10.676908 -0.240639<br>22.C 2.498401 9.815880 2.505920<br>23.H 1.629865 9.765240 3.180887<br>24.H 2.708187 10.877392 2.295358<br>25.H 3.361241 9.401684 3.050372<br>26.C -1.962532 6.212974 4.716675<br>27.H -1.973009 6.224732 5.805444<br>28.C -1.999469 5.052231 3.910342<br>29.H -2.053917 4.023658 4.268208<br>30.C -1.980477 5.463820 2.547301<br>31.H -2.001872 4.807199 1.678834<br>32.C -1.915790 6.875475 2.523070<br>33.H -1.900590 7.491775 1.624973<br>34.C -1.894108 7.370974 3.874034<br>35.C -4.123038 9.301047 4.758528<br>36.H -4.710137 9.050685 3.861581<br>37.H -4.382486 10.326872 5.066777<br>38.H -4.434456 8.612237 5.558917<br>39.C -1.355343 9.621726 5.998900<br>40.H -0.265936 9.646870 5.849761<br>41.H -1.565975 8.915472 6.816887<br>42.H -1.683035 10.622125 6.325466<br>43.C -1.831734 10.369344 3.045828	

44.H -0.754414 10.385093 2.826812<br>45.H -2.129683 11.384234 3.354342<br>46.H -2.363647 10.134392 2.110848<br>47.C 2.865265 5.547056 5.029899<br>48.H 3.790138 5.340963 4.494925<br>49.C 2.416916 6.822231 5.434225<br>50.H 2.940302 7.765797 5.277337<br>51.C 1.170098 6.661346 6.104695<br>52.H 0.577656 7.454757 6.555297<br>53.C 0.852050 5.283773 6.097361<br>54.H -0.031508 4.836673 6.550706<br>55.C 1.892221 4.564118 5.412065<br>56.C 3.818590 2.264969 4.698357<br>57.H 3.941192 1.171593 4.647554<br>58.H 4.578511 2.663404 5.387592<br>59.H 4.026036 2.673928 3.698288<br>60.C 1.809060 1.920527 6.999863<br>61.H 0.802986 2.147794 7.385199<br>62.H 2.537366 2.311993 7.726173<br>63.H 1.915589 0.824620 6.955566<br>64.C 0.818301 1.891283 4.119718<br>65.H -0.214377 2.107133 4.434433<br>66.H 0.948330 0.795986 4.112408<br>67.H 0.932791 2.257829 3.088561	

Table S3. Relative single-point energy difference in kcal/mol between $f^{10}d^1$ and $f^{11}d^0$ in $Ho^{II}(C_5H_4SiMe_3)_3^{1-}$ from different functional results at the PBE//TZP optimized ground-state geometries.

Compound	ΔE(PBE)	ΔE(B3LYP)	ΔE(BHandHLYP)
$f^{10}d^1$	0	0	0
$f^{11}d^0$	-15.1	-1.8	27.1
